# *In vivo* characterization of 3D-printed polycaprolactone-hydroxyapatite scaffolds with Voronoi design to advance the concept of scaffold-guided bone regeneration

**DOI:** 10.3389/fbioe.2023.1272348

**Published:** 2023-10-04

**Authors:** Markus Laubach, Buddhi Herath, Nathalie Bock, Sinduja Suresh, Siamak Saifzadeh, Bronwin L. Dargaville, Jacqui McGovern, Marie-Luise Wille, Dietmar W. Hutmacher, Flavia Medeiros Savi

**Affiliations:** ^1^ Australian Research Council (ARC) Training Centre for Multiscale 3D Imaging, Modelling, and Manufacturing (M3D Innovation), Queensland University of Technology, Brisbane, QLD, Australia; ^2^ Centre for Biomedical Technologies, School of Mechanical, Medical and Process Engineering, Queensland University of Technology, Brisbane, QLD, Australia; ^3^ Department of Orthopaedics and Trauma Surgery, Musculoskeletal University Center Munich (MUM), LMU University Hospital, LMU Munich, Munich, Germany; ^4^ Jamieson Trauma Institute, Metro North Hospital and Health Service, Royal Brisbane and Women’s Hospital, Herston, QLD, Australia; ^5^ Max Planck Queensland Centre (MPQC) for the Materials Science of Extracellular Matrices, Queensland University of Technology, Brisbane, QLD, Australia; ^6^ Biomechanics and Spine Research Group at the Centre of Children’s Health Research, Queensland University of Technology, Brisbane, QLD, Australia; ^7^ Medical Engineering Research Facility, Queensland University of Technology, Chermside, QLD, Australia; ^8^ ARC Training Centre for Cell and Tissue Engineering Technologies, Queensland University of Technology, Brisbane, QLD, Australia

**Keywords:** scaffold, polycaprolactone, hydroxyapatite, Voronoi, 3D printing, scaffold-guided bone regeneration

## Abstract

Three-dimensional (3D)-printed medical-grade polycaprolactone (mPCL) composite scaffolds have been the first to enable the concept of scaffold-guided bone regeneration (SGBR) from bench to bedside. However, advances in 3D printing technologies now promise next-generation scaffolds such as those with Voronoi tessellation. We hypothesized that the combination of a Voronoi design, applied for the first time to 3D-printed mPCL and ceramic fillers (here hydroxyapatite, HA), would allow slow degradation and high osteogenicity needed to regenerate bone tissue and enhance regenerative properties when mixed with xenograft material. We tested this hypothesis *in vitro* and *in vivo* using 3D-printed composite mPCL-HA scaffolds (wt 96%:4%) with the Voronoi design using an ISO 13485 certified additive manufacturing platform. The resulting scaffold porosity was 73% and minimal *in vitro* degradation (mass loss <1%) was observed over the period of 6 months. After loading the scaffolds with different types of fresh sheep xenograft and ectopic implantation in rats for 8 weeks, highly vascularized tissue without extensive fibrous encapsulation was found in all mPCL-HA Voronoi scaffolds and endochondral bone formation was observed, with no adverse host-tissue reactions. This study supports the use of mPCL-HA Voronoi scaffolds for further testing in future large preclinical animal studies prior to clinical trials to ultimately successfully advance the SGBR concept.

## Graphical Abstract

**Figure:**
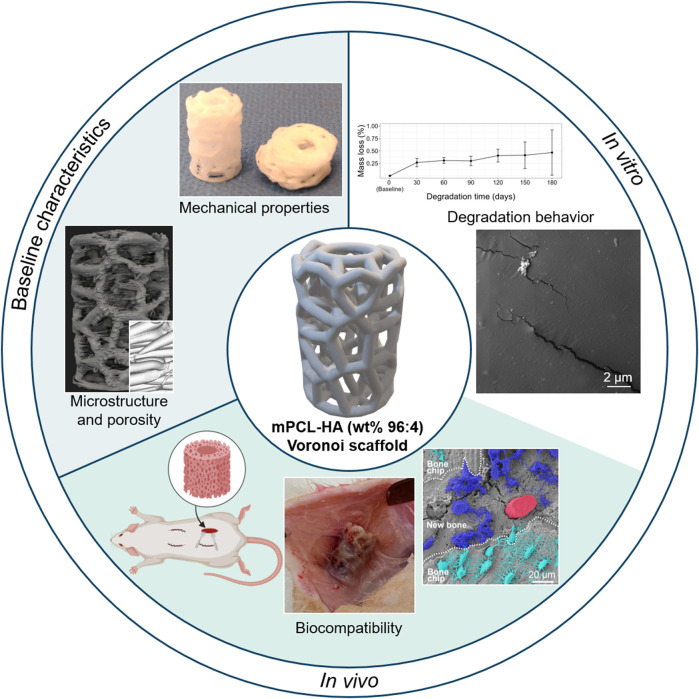
partially created with BioRender.com.

## 1 Introduction

Scaffold-guided bone regeneration (SGBR) is a clinically applied concept that has largely improved the treatment of bone defects in the last decade, as demonstrated by the findings from multiple large animal and clinical studies by our research group and others (Reichert et al., 2012; Cipitria et al., 2013; Berner et al., 2015; Berner et al., 2017; Kobbe et al., 2020; Henkel et al., 2021; Laubach et al., 2022b; Castrisos et al., 2022; Sparks et al., 2023). The SGBR concept is based on the application of biodegradable porous implants (or “scaffolds”) that act as carriers for bone grafts (Hutmacher, 2000). Bone grafts, which typically consist of bone chips, bone marrow, or a combination of both, are often collected from the intramedullary canal of long bones (Laubach et al., 2023b). Before the SGBR concept was established, such bone grafts were transferred from the collection site to the defect site during the same surgery, without the use of scaffolds. As bone grafts have highly osteogenic properties, they facilitate strong bone regeneration at the defect site. The use of bone grafts alone for large bone defects is however associated with inherent shortcomings, namely undesired creeping and premature resorption of the graft. This may result in insufficient bone regeneration and failure to achieve sufficient structural stability required for restoration of full function (Weiland et al., 1984; Masquelet, 2003). To address these challenges, the concept of SGBR relies on incorporating the graft material within slowly-degrading porous scaffolds that act as a supportive structural template for tissue ingrowth. Superior regeneration is enabled by withstanding structural and mechanical forces, necessary for timely physiological remodeling of the transplanted graft. Ultimately, applying the concept of SGBR leads to less graft resorption and better functional bone regeneration (Laubach et al., 2022b).

Biodegradable scaffolds are typically made from natural materials, such as collagen and fibrin, or from synthetic polymers such as polycaprolactone (PCL) (Hutmacher, 2000; Salgado et al., 2004; Hutmacher et al., 2007). The rate of degradation of PCL is relatively slow for scaffolds larger than 1 cm^3^ (>2–4 years *in vivo*) due to its hydrophobic nature and the associated low rate of hydration and subsequent hydrolytic cleavage (Bartnikowski et al., 2019; Laubach et al., 2022b). This implies that bioresorption occurs only after several phases of bone remodeling, after new bone has been established and remodeled to a mature state within the pores of the scaffold (Hutmacher, 2000). However, the osteogenicity of PCL alone is limited, and higher bioactivity is often achieved by using composite materials (Woodruff and Hutmacher, 2010). For SGBR, suitable degradation properties of the scaffold and enhancement of bioactivity are best achieved by incorporating ceramic fillers such as hydroxyapatite (HA) (Bartnikowski et al., 2019), which also increase bone-forming potential *in vivo* (Chuenjitkuntaworn et al., 2010; Ma et al., 2019). Biodegradable composite implants made of PCL-HA are approved by the United States Food and Drug Administration (FDA) for bone defect treatment (DePuy Synthes’ TRUMATCH Graft Cage) (Liodakis et al., 2023); however, these implants do not present with a fully interconnected three-dimensional (3D) pore architecture, which is essential for SGBR (Laubach et al., 2023b).

The capacity for *in vivo* tissue integration and bone formation depends on 3D implant architecture, as the rate and degree of bone ingrowth is determined by the scaffold morphology (Eichholz et al., 2022; Han et al., 2023; Zhou et al., 2023). In particular, efficient mass transfer of oxygen and nutrients to the infiltrating cells depends on timely angiogenesis, which is linked to the scaffold morphology (Serbo and Gerecht, 2013; Collins et al., 2021; Filippi et al., 2023). Key literature, including preclinical and clinical studies, now indicates that the hallmarks for successful application of biodegradable scaffolds in SGBR are high porosity (>70%), a fully interconnected pore network, and a slow biomaterial degradation profile with mass loss below 5% during the first 6 months after surgery (Hutmacher, 2000; Cipitria et al., 2012; Henkel et al., 2021; Laubach et al., 2022b). Examples of 3D designs of scaffolds fulfilling these characteristics are depicted in Supplementary Figure S1, in which they are broadly classified into 4 generations (Generation 1.0–4.0). Generation 1.0–3.0 scaffolds have rectilinear layering patterns and consist of varying the compositions (1.0 = PCL only; 2.0 = PCL composite; and 3.0 = PCL composite with bioactive coating), but these scaffolds do not contain the structural variations that are known to positively affect angiogenesis and successful bone regeneration. With advancements in novel computer aided design (CAD) and additive manufacturing technologies, Generation 4.0 scaffolds were introduced, which have more biomechanically optimized scaffold designs and morphologies (Herath et al., 2021; Basu et al., 2022). One of these complex designs is based on the Voronoi tessellation, where a 3D volume is tessellated into irregular polyhedrons initiated by a set of randomly distributed points, whose edges are connected to form a highly porous network structure (Herath et al., 2021; Herath et al., 2023). The Voronoi design grants the next-generation scaffolds (Generation 4.0) a higher surface to volume ratio and a superior biomimicking of the “trabecular” structure with fully interconnected pores, without compromising on mechanical strength (Liu et al., 2019). Such Generation 4.0 scaffolds, which form an integral part of the further development of the SGBR concept, are characterized by a complex 3D architecture with sufficiently large pores to support their loading with (autologous) bone grafts without compromising their biomechanical properties (Laubach et al., 2023b; Herath et al., 2023). Moreover, in the past, 3D-printed Voronoi scaffolds have been associated with successful bone regeneration attempts using titanium-based biomaterials (Chen et al., 2020; Liang et al., 2022; Lu et al., 2023). However, despite the known relevance of biodegradable PCL-HA as a composite material for bone regeneration (Liodakis et al., 2023), it has not been assessed within the context of 3D-printed SGBR scaffolds with the Voronoi design, which is the objective of this work.

Therefore, to advance the concept of SGBR, we tested Voronoi design based SGBR scaffolds 3D-printed with the FDA-approved medical-grade PCL-HA (mPCL-HA) composite biomaterial (referred to as mPCL-HA Voronoi scaffolds). It is known that bone grafts have excellent osteogenic potential for bone regeneration (Reichert et al., 2011), specifically because they contain growth factors relevant to bone formation (Palta et al., 2014; Laubach et al., 2023a). We thus tested the novel mPCL-HA Voronoi scaffolds in combination with bone chips, bone marrow or both together, as this may be done interchangeably in a clinical setting, depending on patient graft availability and surgeon preference (Laubach et al., 2023a). Our objectives in this study were to 1) perform in-depth *in vitro* and *in vivo* testing to evaluate the physical properties of Generation 4.0 mPCL-HA Voronoi scaffolds, 2) assess the *in vivo* biocompatibility and osteoimmune response of Generation 4.0 mPCL-HA Voronoi scaffolds alone, and 3) in combination with bone grafts. An ectopic bone formation rat model was used for the *in vivo* study as it allowed the implantation of a combination of larger 3D-printed mPCL-HA Voronoi scaffolds and clinically-relevant fresh bone grafts from sheep. Intramedullary harvesting devices routinely used in the clinic were used to harvest bone grafts from sheep. The combination of scaffolds and fresh bone graft is based on the SGBR concept and we anticipate that this approach represents the necessary prerequisites toward larger animal studies and future clinical trials to ultimately achieve the successful translation of mPCL-HA Voronoi scaffolds into clinical practice.

## 2 Materials and methods

### 2.1 Scaffold design and manufacturing

Scaffolds were designed in-house based on the Voronoi tessellation (Chen et al., 2020), where a region can be divided into discrete cells based on a randomly populated set of points known as “seed points” (Herath et al., 2021). The design process was undertaken using the software suite Rhinoceros 3D & Grasshopper (Robert McNeel & Associates, Seattle, United States). The Voronoi lattice structure was derived by tessellating a uniform tubular cylinder of an outer diameter of 10 mm, an inner diameter of 4 mm, and a height of 15 mm, with 125 seed points and a strut diameter of 1 mm. The final Voronoi scaffold design was exported in a stereolithography (STL) file format and shared with the manufacturer. The 3D mesh model is shown in Supplementary Figure S2. Scaffolds were additively manufactured using a composite material of mPCL with 4% HA (Evonik Industries AG, Essen, Germany) under ISO 13485 certification by BellaSeno GmbH (Leipzig, Germany).

### 2.2 Characterization of 3D-printed mPCL-HA Voronoi scaffolds

#### 2.2.1 Evaluation of morphology and porosity

All micro-computed tomography (μCT) scanning was done using a Scanco Medical AG μCT 50 scanner (Scanco, Brüttisellen, Switzerland) at an energy of 55 kVp and current of 145 μA, with a 0.1 mm aluminum filter. All samples dedicated for evaluation of morphology and porosity were scanned in air and wrapped in low density foam to prevent movement. The manufacturing quality was evaluated by quantitative assessment of the fabrication accuracy in terms of the distribution of HA particles and filament diameters by scanning the scaffolds (*n* = 2) at high resolution with an isotropic voxel size of 1.2 μm^3^ (integration time = 1,500 ms).

A quantitative assessment of scaffold porosity was obtained by scanning pristine scaffolds (*n* = 8) with an isotropic voxel size of 14.8 μm^3^ (integration time = 300 ms) and then conducting 3D reconstructions to obtain the as-manufactured geometry (referred to as µCT models) using the Amira 2020.2 software (Thermo Fischer Scientific, United States). The reconstructed µCT model was imported to Rhinoceros 3D & Grasshopper software, and a uniform tubular cylinder was aligned to envelope the model and compared with the designed Voronoi CAD model (referred to as CAD model). The volumes of the tubular cylinder and the µCT model were calculated, and, finally, the porosity was derived as follows (Eq. 1):

$$Porosity\;\left(\%\right)=\frac{VE-VS}{VE}\times 100\%$$ (1)

where VE is the volume of the tubular cylindrical envelope and VS is the volume of the segmented µCT model.

Scaffold morphology (*n* = 7) was further studied by scanning electron microscopy (SEM). Scaffold surfaces were platinum sputtered at 4 nm coated (Leica ACE600 high vacuum sputter coater, Leica Microsystems, Australia) and then imaged with a TESCAN MIRA 3 high-resolution analytical SEM (Tescan, Brno, Czech Republic) at an accelerating voltage of 5.0 kV, a beam intensity of 8.0, and constant working distance of 8.0 mm.

#### 2.2.2 Compression testing

Scaffolds (*n* = 6) were subjected to uniaxial compressive testing to determine their compressive properties, specifically their Young’s modulus and yield strength. The samples were immersed in a 1X phosphate buffered saline bath (PBS, Sigma-Aldrich) under simulated physiological conditions (37°C), and unconfined tests were performed with a strain rate of 0.1 mm/s using a 2 kN load cell on an Instron model 5567 instrument (Melbourne, Australia). The experimental setup is shown in Supplementary Figure S3. We used the calculated slope of the initial linear region of the fitted stress-strain curve as the elastic modulus (Young’s modulus). Yield strength was measured at 0.2% offset strain.

### 2.3 *In vitro* degradation kinetics

The *in vitro* hydrolytic degradation was assessed by immersing the mPCL-HA 3D-printed scaffolds in 1X PBS, as described in comparative studies investigating the hydrolytic degradation of PCL-based materials (Abdal-hay et al., 2020). For the different degradation analyses, the specimens (*n* = 7) were individually introduced into 15 mL Falcon tubes containing 10 mL sterile 1X PBS at pH 7.4 with 1% penicillin-streptomycin, closed to avoid evaporation, and maintained statically at 37°C. The solution was fully replaced once every 30 days. At different times (30, 60, 90, 120, 150, and 180 days), the samples were washed three times with deionized water and dried overnight in an oven at 37°C under vacuum. Week 0 was defined as the baseline, and at each time point the morphological characteristics, mass loss, and thermal properties were determined. With the exception of the week 0 samples (baseline), degraded samples were collected at different time points and used for the analyses described hereafter.

#### 2.3.1 Morphology with scanning electron microscopy

The samples were trimmed with a scalpel and their surface and morphology were examined by SEM, as previously described in Section 2.2.1.

#### 2.3.2 Mass variation

Mass loss from individual scaffolds was determined as previously described (Lam et al., 2009) by calculating the difference in mass at baseline and at each time point, measured with scales (0.1 mg resolution), as follows (Eq. 2):

$$Mass\;loss\;\left(\%\right)=\frac{Mi-Mf}{Mi}\times 100\%$$ (2)

where *Mi* is the initial mass, and *Mf* is the final mass.

#### 2.3.3 Gel permeation chromatography

Gel permeation chromatography (GPC) was used to determine the weight average (M_w_) and number average (M_n_) relative molecular weight and dispersity of the mPCL-HA scaffolds. Sections of the mPCL-HA scaffolds were cut and dissolved in tetrahydrofuran (THF) at a concentration of 1 mg/mL. All samples were passed through 0.22 μm PTFE membrane filters. Measurements were conducted on a PSS SECurity2 system consisting of a PSS SECurity Degasser; PSS SECurity TCC6000 column oven (35°C); PSS SDV column set (8 × 150 mm; 5 μm precolumn; 8 × 300 mm; 5 μm analytical columns; and 100,000 Å, 1,000 Å, and 100 Å); and an Agilent 1260 Infinity isocratic pump, Agilent 1260 Infinity standard autosampler, Agilent 1260 Infinity diode array and multiple wavelength detector (A: 254 nm, B: 360 nm), and Agilent 1260 Infinity refractive index detector (35°C). HPLC-grade THF—stabilized with BHT—was used as an eluent at a flow rate of 1 mL/min. Narrow-disperse linear poly (methyl methacrylate) (M_n_: 202 g/mol to 2.2 × 10^6^ g/mol) standards (PSS ReadyCal) were used as calibrants. Molecular weight and dispersity analyses were performed using PSS WinGPC UniChrom software (version 8.2).

#### 2.3.4 Thermal property analysis

The thermal properties of the mPCL-HA composite scaffolds were investigated by differential scanning calorimetry (DSC), as described previously (Mohseni et al., 2018). Briefly, DSC was conducted using a Q100 DSC apparatus (TA Instruments, Newcastle, DE, United States) in aluminum crucibles in an argon atmosphere to study the thermal properties, such as glass transition temperature, melting temperature, and crystallinity of the 3D-printed scaffolds. The sample mass was 5–10 mg. The following temperature conditions were used: heating from 0°C to 100°C at a rate of 10°C/min, an isothermal segment at 100°C for 5 min, cooling to 0°C at a rate of 10°C/min, and an isothermal segment at 0°C for 5 min. Analysis was performed on the thermographs, utilizing Universal Analysis 2000 software (TA Instruments, Newcastle, DE, United States). The melting point and the heat of fusion (the heat of melting) were determined to calculate the polymer crystallinity. A reference of 135 J/g was used for 100% crystalline PCL (Crescenzi et al., 1972), and the degree of crystallinity (X_c_ %) of the polymer scaffold was calculated as follows (Eq. 3):

$$Xc\;\left(\%\right)=\frac{∆\text{Hm}}{∆\text{Hmo}}\times 100\%$$ (3)

where ∆Hm and ∆Hmo are the enthalpies of melting of the sample and the hypothetical polymer that is 100% crystalline, respectively.

The thermal stability of the fabricated scaffolds was investigated by thermogravimetric analysis using a TGA Q50 instrument from ambient temperature to 600°C at a heating rate of 10°C/min under nitrogen gas flowing at 60 mL/min.

### 2.4 Animal experiments

#### 2.4.1 *In vivo* study

Biocompatibility and osteoimmunomodulatory capacity of the scaffolds were assessed using a rat ectopic bone formation model. Ethical approval was obtained from the Queensland University of Technology (QUT) Animal Ethics Committee (UAEC) (Ethics Approval Numbers 2000000592 and 2000000593). All animal surgeries were performed at the QUT Medical Engineering Research Facility (MERF) at the Prince Charles Hospital campus (Chermside, Queensland, Australia). The study was conducted in accordance with the requirements of the Australian Code of Practice for the Care and Use of Animals for Scientific Purposes, and the ARRIVE 2.0 guidelines (Animal Research: Reporting of *In Vivo* Experiments) (Percie du Sert et al., 2020) were followed.

In this study, two 12-week-old male CBH-rnu/Arc (nude) rats with body weight (BW) of 217–260 g were purchased from the Animal Resources Centre (Canning Vale, Western Australia) and housed in individually ventilated double decker cages (Techniplast, GR 1800) in a pathogen-free and temperature-controlled environment. CBH-rnu/Arc (nude) rats are homozygous for the Foxn1^nu^ mutation, which results in failure of thymus formation (dysgenesis of the thymus). This leaves no place for CD4^+^ and CD8^+^ T cells to differentiate and mature, making these homozygote rats T cell deficient (Kyriakides et al., 2022). The rats were allowed to acclimatize for a minimum of 1 week before commencing the experimental procedures and had access to sterile food and water *ad libitum*.

The rats received scaffolds alone (using the scaffold by itself without any bone grafts) and also scaffolds loaded with different types of ovine bone graft (1.0 g per scaffold). Bone graft was freshly harvested under sterile conditions from one female Merino sheep (55 kg BW, 1–2 years of age). Three different methods of harvesting bone graft from the intramedullary canal of the sheep’s femur or tibia were used: the Reamer-Irrigator-Aspirator (RIA) 2 system to harvest bone graft mix of bone chips and bone marrow, an aspirator to collect bone marrow, and application of a reaming-aspiration (R-A) method to harvest bone chips following bone marrow removal (Supplementary Figure S4). Table 1 lists the details of the harvesting methods, associated bone graft types, and experimental groups. Fibrin glue (160 μL, TISSEEL Fibrin Sealant, Baxter Healthcare International) was added to the different types of bone graft and then loaded onto the scaffolds, which had been sterilized with 80% (v/v) ethanol (evaporation method) (Lahr et al., 2020). All procedures, including scaffold preparation and perioperative procedures, were conducted in biosafety cabinets. Details for the setup of the biosafety cabinet are illustrated in Supplementary Figure S5.

**TABLE 1:** Experimental groups and bone graft material types used in rat ectopic bone formation study. The material was mixed with fibrin glue (160 μL) and loaded onto the mPCL-HA Voronoi scaffolds (*n* = 2).

Group	Harvesting device/method	Bone graft material type
Sc	Scaffold alone	
ScRIA2	RIA 2 system	Bone chips and bone marrow
ScA	Aspirator	Bone marrow
ScRA	R-A method	Bone chips

mPCL-HA, medical-grade polycaprolactone-hydroxyapatite; RIA 2 system, Reamer-Irrigator-Aspirator 2 system; R-A method, reaming-aspiration method; Sc, scaffold.

The rat surgical procedure was adapted from Sabini et al. (2000). General anesthesia was induced and maintained with isoflurane (2%–4%) in oxygen. Subcutaneous (sc.) buprenorphine (0.05 mg/kg BW) and meloxicam (1 mg/kg BW) were administered for preemptive analgesia. Prophylactic antibiotic cefazolin (sc., 20 mg/kg BW) was given once preoperatively and for 2 days postoperatively. In total, four different types of constructs including scaffold alone or in combination with different types of fresh ovine bone grafts, as outlined in Table 1, were implanted in subcutaneous pockets created on the rat dorsum. Supplementary Figure S6 illustrates the scaffold preparation and (peri)operative procedures for anesthesia and surgery. Tramadol in drinking water (25 mg/L) was given for 5 days after surgery for postoperative analgesia. The rats were euthanized with CO_2_ asphyxiation 8 weeks post-implantation, as per National Health and Medical Research Council (NHMRC) guidelines.

#### 2.4.2 Faxitron and μCT imaging of the specimens

Post-euthanasia, the animals were imaged inside a portable high-resolution X-ray cabinet (Faxitron MX-20, Faxitron Bioptics LLC, United States). Subsequently, the specimens were retrieved and fixed in 4% paraformaldehyde for 4 days before transferring to 70% (v/v) ethanol until needed for further analyses. All the specimens underwent µCT scanning with an isotropic voxel size of 17.2 μm^3^ (integration time 800 ms); they were assessed at a threshold of 340/1,000, a Gaussian filter width of 0.8, and gauss filter support of 1, and the total bone volume was calculated using the SCANCO proprietary scan evaluation software after segmentation.

#### 2.4.3 Histology and immunohistochemistry

Fixed samples were cut longitudinally in two parts using an EXAKT 310 Diamond Band Saw (EXAKT Apparatebau GmbH & Co. KG, Norderstedt, Germany) to allow histological assessment of decalcified paraffin embedded samples by immunohistochemistry (IHC) as well as undecalcified samples embedded in resin.

For histological analysis, the samples were decalcified for 1 week in 10% EDTA pH 7.4 at 37°C using a KOS rapid microwave lab station (ABACUS, Brisbane, Australia). The decalcification process was verified by qualitative X-ray analysis. Samples were serially dehydrated in ethanol in an automated Excelsior ES tissue processor (Excelsior ES, Thermo Scientific, Franklin, MA, United States) and embedded in paraffin. Sections of 5 μm thickness were cut, collected onto polylysine-coated microscope slides, and then dried at 60°C for 16 h. A subset of the slides was stained with hematoxylin and eosin (H&E) using a Leica Autostainer XL (Leica Biosystems, Nussloch, Germany). Histological staining and immunohistochemistry were performed according to the protocol previously established by our group (Sparks et al., 2020). The primary antibodies specific to the osteogenic and osteoimmunomodulatory markers used for this study and the protocol specifications are provided in Supplementary Table S1. Stained slides were scanned using a 3DHistech Scan II Brightfield slide scanner (3DHistech, Budapest, Hungary) at ×20 objective with a spatial resolution of 0.27 mm.

Undecalcified samples were embedded in resin, as described by Sparks et al. (2020, for histomorphological evaluations. The embedded tissues were sectioned longitudinally at 200 μm using an EXAKT 310 Diamond Band Saw and ground to 30 μm for histological analysis and to 100 μm for SEM analysis, using an EXAKT 400CS micro grinder (EXAKT Apparatebau GmbH & Co. KG, Norderstedt, Germany).

The 30 μm ground resin sections were stained with modified Goldner’s Trichrome following standard laboratory protocols (Sparks et al., 2020). The stained sections were imaged using a Carl Zeiss microscope (ZEISS Axio Imager 2, Carl Zeiss Microscopy, NY, United States).

The 100 μm thickness resin sections were ground sequentially with sandpaper to produce a mirror-finish surface. The slides were then etched with 37% phosphoric acid (Ajax Finechem, Albany, NZ, cat. no. AJA371-2.5LPL) for 3 s, washed in running tap water for 5 min, and surface etched in 12.5% sodium hypochlorite (Ajax Finechem, cat. no. AJA82-500G) for another 5 min, followed by washing in running tap water for 5 min. The slides were dried overnight at room temperature. Subsequently, the ground sections were platinum sputtered at 4 nm (Leica ACE600 high vacuum sputter coater, Leica Microsystems, Australia) and then imaged with a TESCAN MIRA 3 high-resolution analytical SEM (Tescan, Brno, Czech Republic) at an accelerating voltage of 5.0 kV, a beam intensity of 8.0, and a constant working distance of 8 mm.

### 2.5 Statistical analyses

The experimental *in vitro* data were analyzed using *R* programming software (version 4.0.2; R Foundation for Statistical Computing, Vienna, Austria) with RStudio, version 1.3.1073 (RStudio Inc., Boston, MA, United States). Values of *p* < 0.05 were considered statistically significant. The results are shown as the mean ± standard deviation (SD) and plotted using *R*. The *R* package “lme” was used to fit a generalized linear model (GLM) to predict the respective degradation parameters with the individual time points for mass loss, molecular weight, and crystallinity (time point as fixed factor). The statistics for this study were chosen in accordance with the guidelines provided by biostatisticians from the Research Methods Group of the QUT.

## 3 Results

### 3.1 3D printing homogeneity, porosity, and mechanical characteristics of scaffolds

**FIGURE 1:**
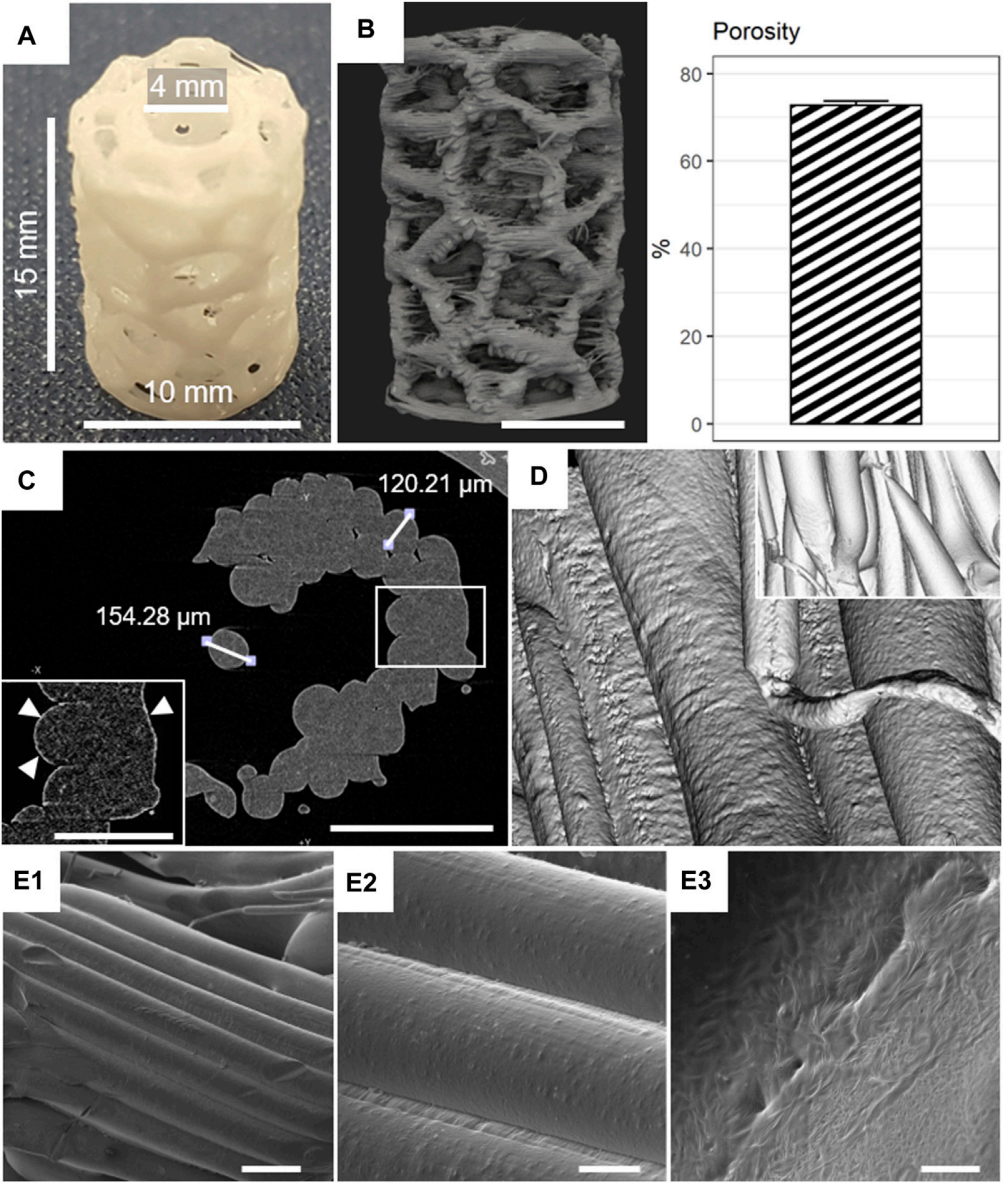
Morphological characterization of mPCL-HA Voronoi scaffolds. **(A)** Macroscopic image. **(B)** Micro-computed tomography (µCT) rendering and corresponding porosity (mean +/- SD shown, *n* = 8). **(C)** Assessment of HA distribution (white triangles). **(D)** µCT rendering of filaments and date reconstruction (inset). **(E1–E3)** Scanning electron microscopy (SEM) images of filaments at low (250×, E1), medium (1,000×, E2), and high (10,000×, E3) magnifications. Scale bars: B, 5 mm; C, 500 μm; C inset, 250 μm; E1, 200 μm; E2, 50 μm; E3, 5 μm.

A representative mPCL-HA scaffold with the Voronoi design after printing is shown in Figure 1A. A reconstructed μCT scan showed adequate reproduction of the desired geometry by the 3D printing process (Supplementary Video S1). In Figure 1B, a fully interconnected strut network with high scaffold porosity of 72.8% ± 0.94% was observed, which is slightly different compared to the expected CAD model porosity of 63.3% (Supplementary Figure S7A). Supplementary Figure S7B shows the workflow for deriving the depicted scaffold porosity. High-resolution µCT did not identify any distinct HA particles (i.e., particles >2 µm in diameter); however, a bright rim in the periphery of the scaffold filaments suggests a more decentralized sub-micrometer powder particle distribution toward the edges of the struts within the mPCL-HA composite (Figure 1C). The filament diameter was approximately 120–155 µm (Figure 1C)—homogenous in size and well connected (Figure 1D). Scanning electron microscopy of the native samples showed high printing accuracy with appropriate filament integrity (Figures 1E1–E3). The mechanical properties of the mPCL-HA scaffolds with Voronoi structure resulted in a Young’s modulus of 11.80 ± 0.35 MPa and a yield strength of 0.76 ± 0.03 MPa. Supplementary Video S2 shows a representative mechanical compression test of the mPCL-HA Voronoi scaffolds. Based on these results, the Generation 4.0 mPCL-HA Voronoi scaffolds produced here were deemed suitable for subsequent *in vitro* and *in vivo* testing.

### 3.2 *In vitro* degradation analysis

**FIGURE 2:**
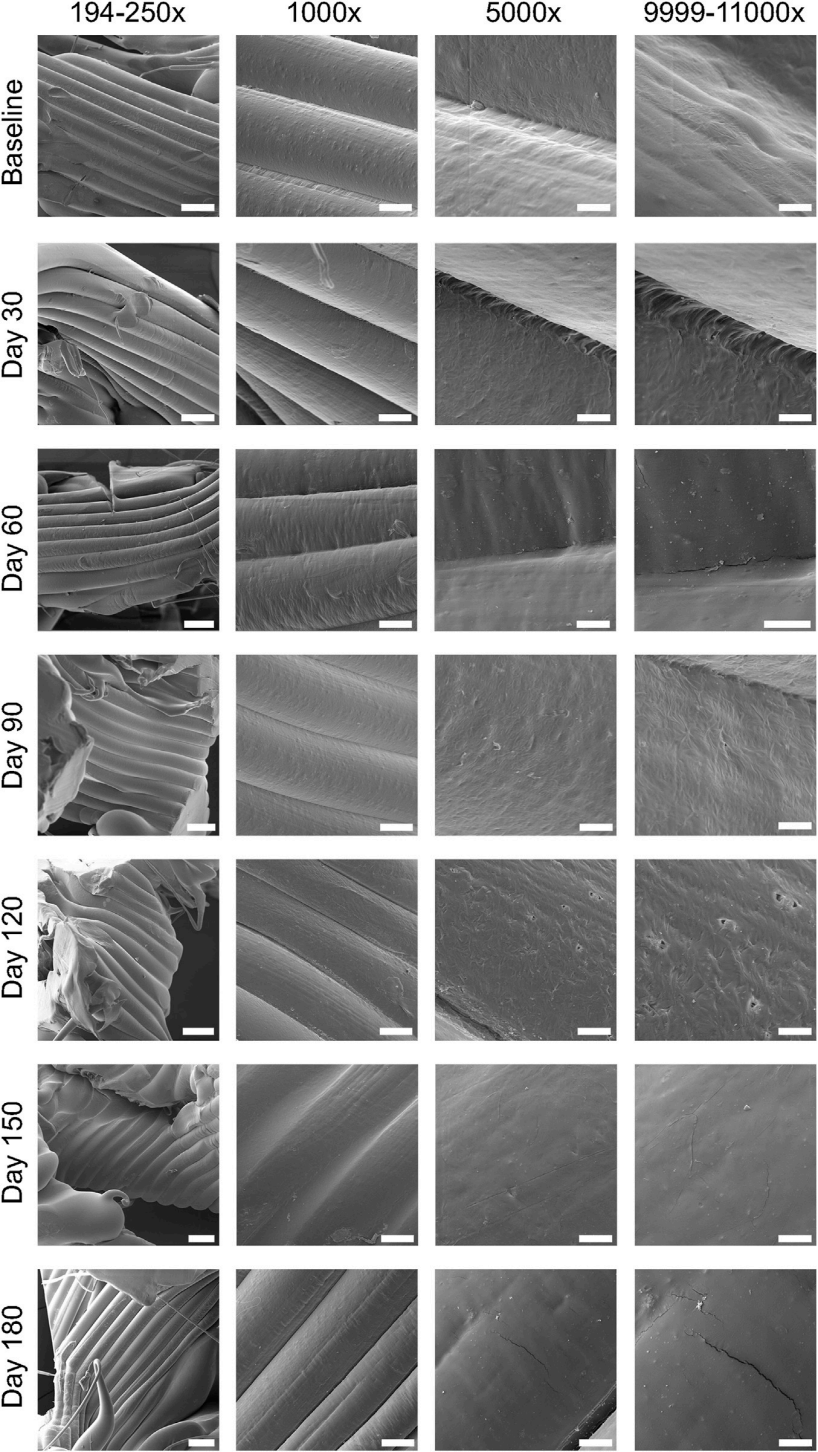
Assessment of surface integrity using scanning electron microscopy (SEM) images obtained during the 180-day period of *in vitro* degradation analysis of mPCL-HA scaffolds, which were kept in 1X PBS at 37°C throughout the follow-up period. Scale bars: 194–250×, 200 μm; 1,000×, 50 μm; 5,000×, 10 μm; 9,999–11,000×, 5 μm.

**FIGURE 3:**
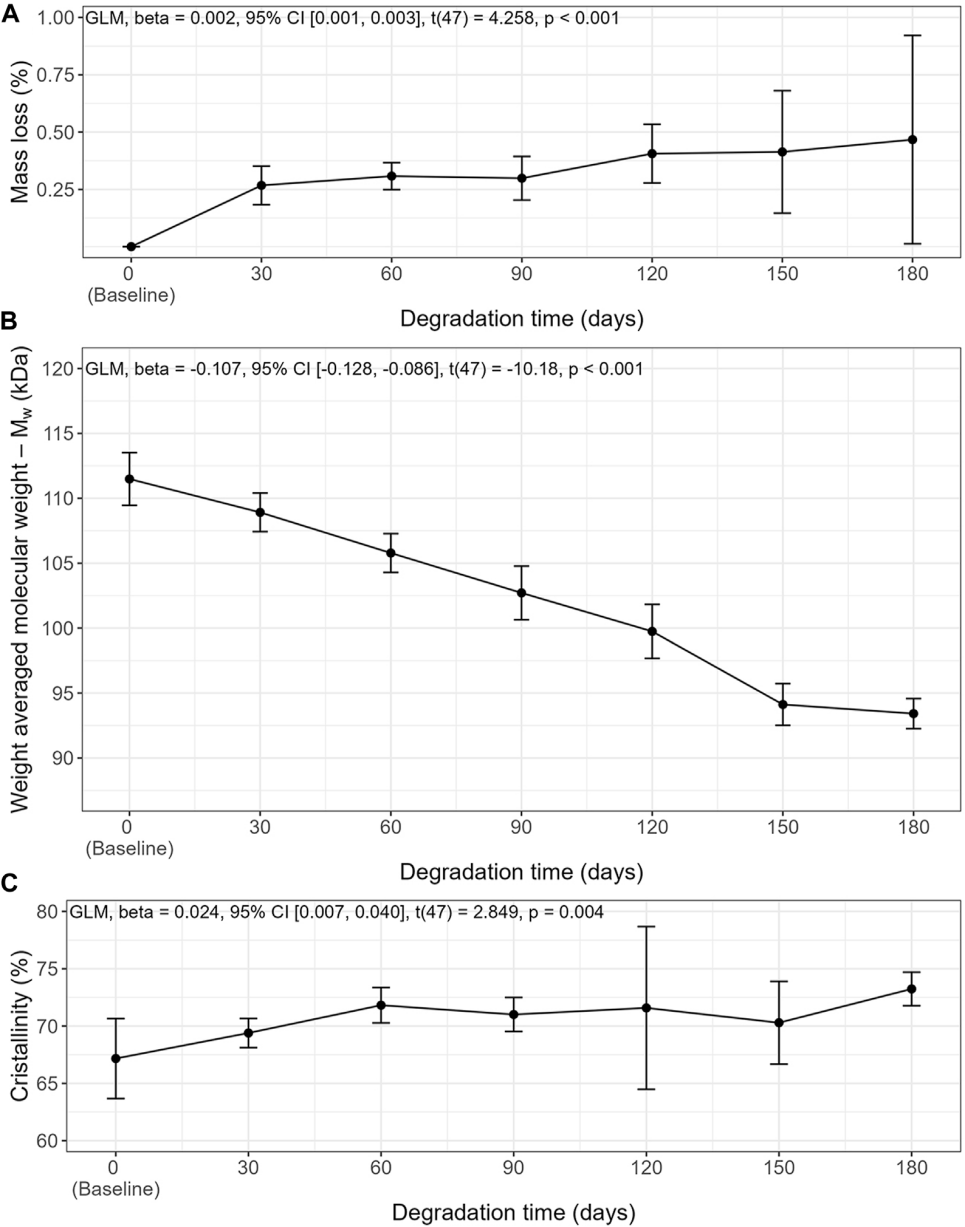
*In vitro* degradation characteristics of mPCL-HA scaffolds with the Voronoi design over time in PBS at 37°C. **(A)** Mass Loss, **(B)** Weight averaged molecular weight, **(C)** crystallinity. Means ± SD, *n* = 7.

Slow degradation of the 3D-printed mPCL-HA Voronoi scaffolds, which were kept under simulated physiological conditions, was observed over a period of 180 days (Figures 2, 3). Scanning electron microscopy imaging indicated surface changes over time, but these minor changes did not disrupt surface integrity. Instead, small, isolated cracks indicated the occurrence of minor erosion processes, with only a slight progression over time within the follow-up period of 180 days (Figure 2). Furthermore, statistical analysis indicated that the effect of time was statistically significant and positive, particularly between baseline and 30 days, where the mass loss was observed to be more prominent compared to the other time points. However, the mass loss between baseline and the 180-day follow-up (0.47% ± 0.46%) was negligibly small (Figure 3A), although it is to be noted that variability increased at each time point. Gel permeation chromatography analysis also showed little reduction in the average molecular weight (M_w_) from baseline (111.48 ± 5.4 kDa) to 180 days (93.42 ± 3.06 kDa), although the effect was statistically significant and can be described as linear (Figure 3B). The plot of number average molecular weight (M_n_) is shown in Supplementary Figure S8. A statistically significant positive effect of time was observed for crystallinity, which increased particularly between baseline and day 60 and between day 150 and day 180 (Figure 3C). However, the overall difference between baseline (67.2% ± 3.5%) and 180 days (73.2% ± 1.5%) was, as expected, very small. Additional information on dispersity index is shown in Supplementary Figure S9.

### 3.3 *In vivo* rat ectopic bone formation model

A standardized operating procedure was followed for scaffold preparation, with and without additional bone graft loading, as per Table 1. Both rats endured the operation and the recovery phase without any relevant complications and reached the protocol end point after 8 weeks.

### 3.4 *In situ* radiographic and macroscopic assessment of specimens

**FIGURE 4:**
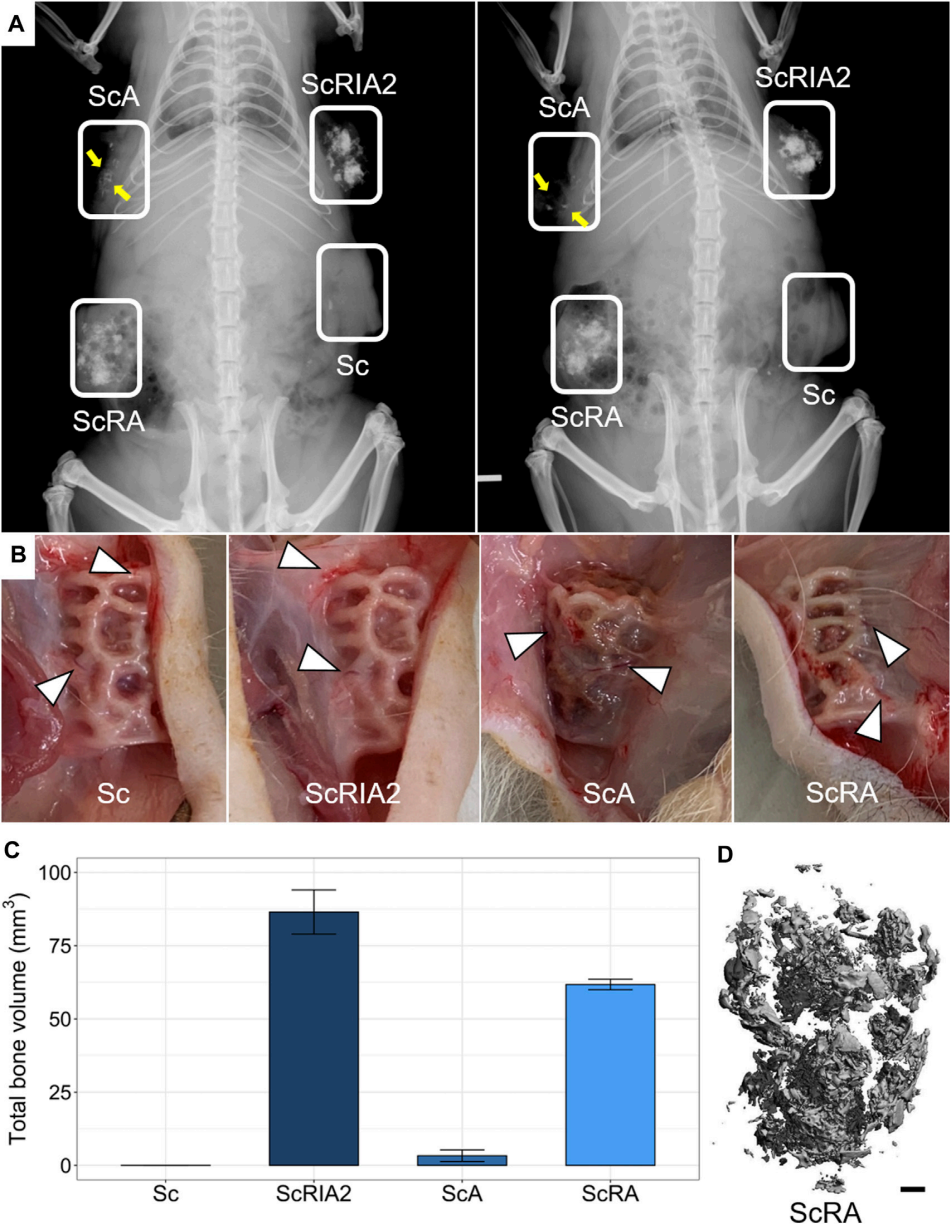
Specimen radiographic, macroscopic and µCT imaging assessments *in situ*. **(A)** High-resolution X-rays. **(B)** Scaffold integration and blood vessel ingrowth from surrounding tissue (indicated with triangles in B). **(C)** Total bone volume quantifications from μCT imaging data (mean ± SD, *n* = 2). **(D)** Representative reconstructed μCT image. Scale bar: D, 1 mm.

High-resolution X-ray imaging of scaffolds *in situ* after euthanasia showed higher radio-opacity in the groups that received bone chips and bone marrow (ScRIA2 group) or bone chips alone (ScRA group) than in the groups with bone marrow alone (ScA group) or with the scaffold alone (Sc group) (Figure 4A). The radio-opacity in different groups indicated a homogeneous distribution of bone graft. Notably, the ScA group showed some opacity that followed the 3D scaffold network architecture (Figure 4A—yellow arrows). The Sc group, by contrast, was radiolucent, with no radiographic signs of new bone formation. Following X-ray imaging, the surgical sites were accessed and the specimens assessed *in situ*. Macroscopically, no overt inflammation or excessive fibrotic tissue layers surrounding the specimens was observed. Moreover, all experimental groups showed appropriate integration into the surrounding tissues, including macroscopically visible ingrowth of blood vessels (Figure 4B). Quantification of the mineralized tissue (total bone volume) using μCT imaging confirmed the observations of high-resolution X-ray imaging (Figure 4C). For the Sc group, very little total bone volume was observed (both 0.0004 mm^3^). Scaffolds loaded with bone marrow (ScA group) showed the lowest mean total bone volume of 3.32 ± 1.97 mm^3^ of all the bone graft test groups. Scaffolds loaded with bone chips and bone marrow (ScRIA2 group) elicited the largest mean total bone volume of 86.48 ± 7.53 mm^3^, while scaffolds loaded with bone chips only (ScRA group) were next with 61.74 ± 1.79 mm^3^. The homogenous distribution of the bone graft is shown in a representative segmented μCT image (Figure 4D—ScRA group) as well as in example (reconstructed) μCT scans of ScRIA2 group (Supplementary Video S3) and ScRA group (Supplementary Video S4).

### 3.5 Immunohistochemistry using osteoimmunomodulatory and osteogenic markers

**FIGURE 5:**
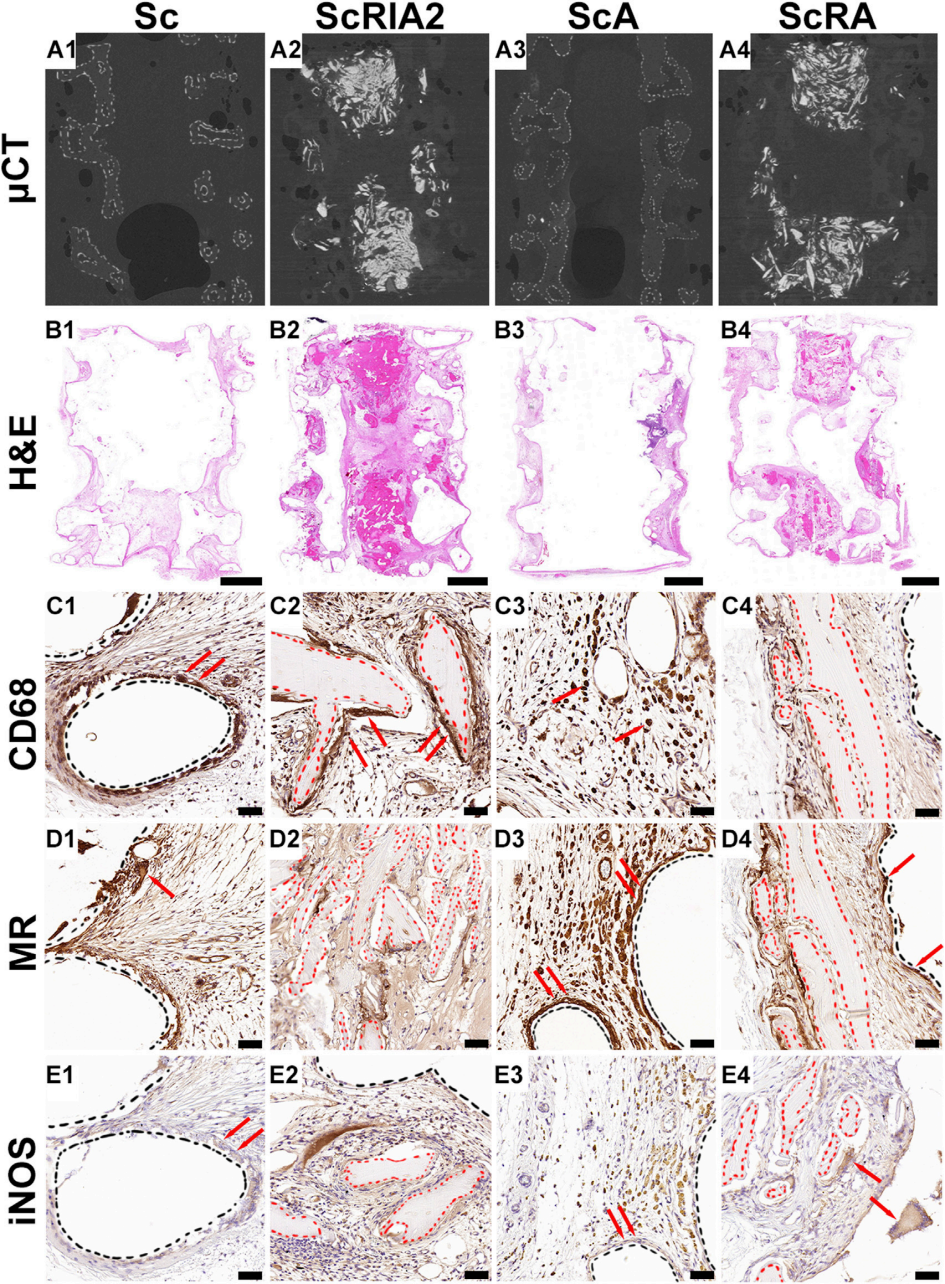
Micro-computed tomography (µCT) and histological and immunohistochemical analysis (osteoimmunomodulatory markers) of explanted specimens. **(A1–A4)** Three-dimensional reconstruction of the µCT data of the sample groups after specimen collection. **(B1–B4)** H&E overview and **(C1–E4)** IHC osteoimmunomodulatory markers. Cluster of differentiation 68 (CD68); inducible nitric oxide synthase (iNOS); Hematoxylin and eosin (H&E); Mannose receptor (MR). Scale bars: B1–B4, 2,000 μm; C1–E4, 50 µm.

Overall, the homogenous distribution of bone graft is shown in representative μCT images (Figures 5A1–A4), and the H&E images of the scaffolds showed good integration within the host tissue (Figures 5B1–B4). The cellular responses around the mPCL-HA scaffold struts (Figures 5C1, C4, D1, D3, D4, 5E1–5E3—black dashed lines) and around the bone graft (Figures 5C2, C4, D2, D4, E2, E4—red dashed lines) were similar for all groups. Both CD68 and Mannose receptor (MR) reactivity were more prominent at a single layer of cells lining the surface of the scaffold struts, at osteoclasts lining the surface of the graft fragments, and also at cells within the tissue around the same areas (Figures 5C1–C3, D1, D4—red arrows). However, there were a few giant cells weakly reactive for nitric oxide synthase (iNOS), especially at the cells lining the outer surface of scaffold struts as well as the bone graft fragments (Figures 5E1, E3, E4—red arrows).

**FIGURE 6:**
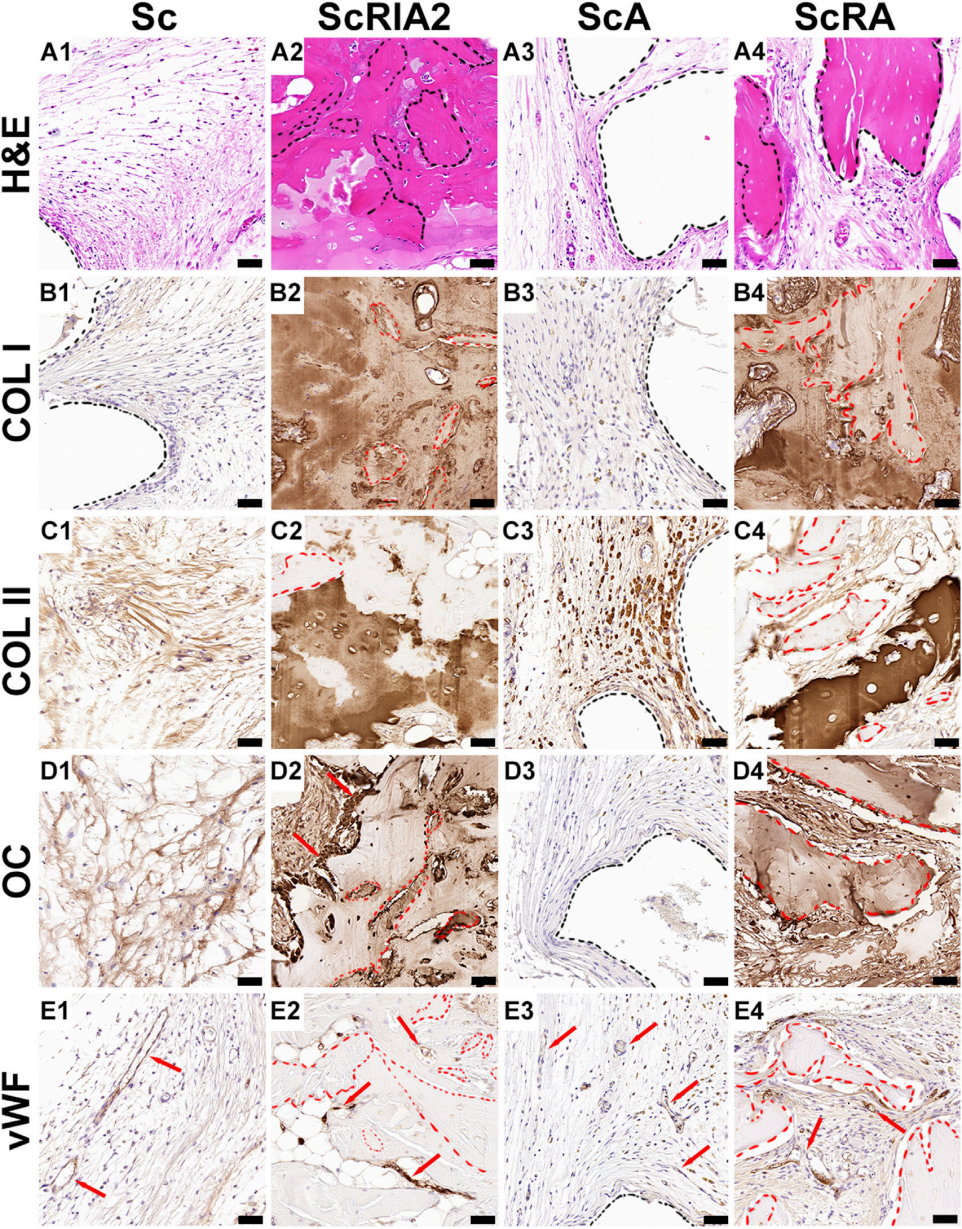
Histological and immunohistochemical analysis (osteogenic markers) of explanted specimens. **(A1–A4)** H&E high magnification and **(B1–E4)** IHC osteogenic markers. Collagen type I (COL I); Collagen type II (COL II); Hematoxylin and eosin (H&E); Osteocalcin (OC); Van Willebrand factor (vWF). Scale bars: A1–E4, 50 µm.

Good tissue formation (Figures 6A1, A3) and integration of bone chips (Figures 6A2, A4—black dashed lines) throughout the scaffold strut architecture (Figures 6A1, A3—black dashed line) was further confirmed on the H&E images with high magnification (Figures 6A1–A4). The Sc group as well as the ScA group showed sparse collagen fiber formation aligning parallel to the outer surface of the scaffold wall (Figures 6A1, A3), and this was further confirmed by the lack of collagen type I (COL I) deposition (Figures 6B1, B3) at scaffold surface architecture (Figures 6B1, B3—black dashed line). Distinguishable stages of mineralization of the extracellular bone matrix could be seen through COL I stain reactivity within the ScRIA2 and ScRA groups (Figures 6B2, 6B4). The same groups presented localized areas of bone remodeling (woven bone), which were heavily stained for COL I and less reactive at the bone chip remnants (Figures 6B2, B4—red dashed lines). These fragments of the original bone graft (Figures 6C2, C4—red dashed lines), identified by the presence of islands of hypermineralized cartilage, were positively stained for collagen type II (Figures 6C2, C4), suggesting osteochondral bone formation (Li et al., 2022). Nevertheless, the bone chips positively stained for osteocalcin (OC), a late osteogenic marker of bone formation, expressed by early osteocytes, as well as at bone lining osteoblasts at the surface of the bone chips (Figure 6D2—red arrows; Figure 6D4). Fragments of the original bone chips (Supplementary Figures S10A, B—red arrows) and an osteon embedded with the new forming bone tissue were visibly distinguishable by H&E staining (Supplementary Figures S10C–E—yellow dotted line). Yet, the intervening space between bone chip fragments was bridged with newly forming bone and was clearly demarcated as depicted by OC staining (Supplementary Figures SF–J—red arrows). Although some of the original architecture of the bone chip fragments implanted were still apparent and empty lacunae were observed, active bone formation could be recognized by the presence of viable osteocytes, osteoclastic activity, osteoid seams (Supplementary Figures S11B–E) and osteoblasts lining the periosteal surface of the graft fragments (Figures 6D2, D4—red dashed lines), and the formation of small marrow cavities (Figure 6E2). The new tissue formed was well vascularized, as demonstrated by the von Willebrand factor positive staining at the endothelial wall of blood vessels for all groups (Figures 6E1–E4—red arrows).

### 3.6 Histomorphometric analysis using modified Goldner’s trichrome staining and SEM

**FIGURE 7:**
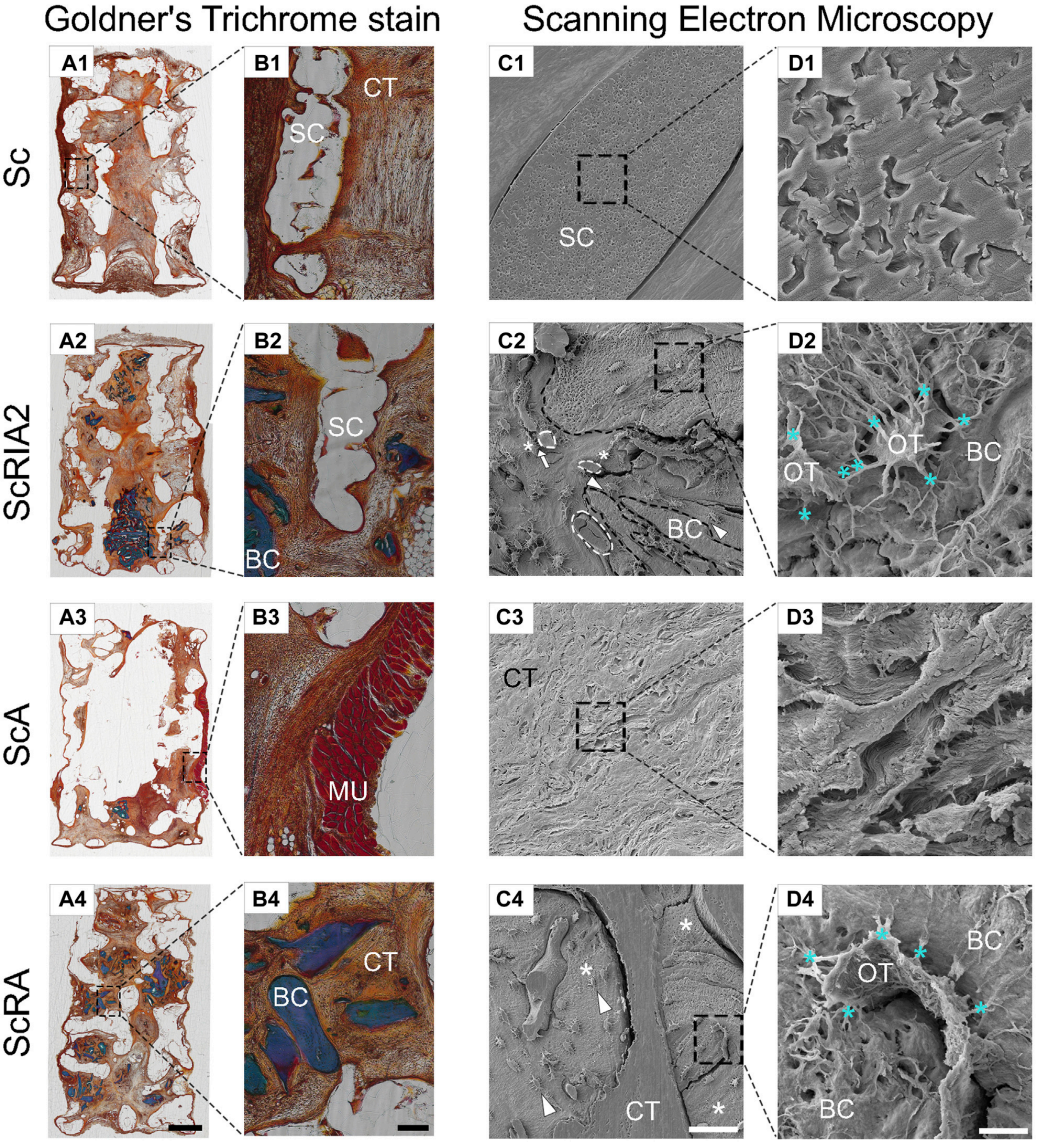
Modified Goldner’s trichrome staining and scanning electron microscopy (SEM) analysis of the mPCL-HA Voronoi scaffolds without and with loading of different types of bone graft. **(A1–B4)** An overview of undecalcified sections is shown with Goldner’s trichrome staining showing good tissue integration throughout all experimental groups. **(C1–D4)** Scanning electron microscopy images following resin cast etching showing the ultrastructural morphology of scaffold-bone graft constructs. Notably, direct contact is observed between blood vessels and cells that are shaped like osteoblastic osteocytes [indicated by arrow in **(C2)**], which in turn may indicate active facilitation of new bone formation. Further, osteoblastic osteocytes next to bone fragments [bone fragments are indicated with black dashed lines in **(C2)**] also connect with those in the newly formed bone, thereby facilitating connectivity with the surrounding connective tissue [white triangles in **(C2,C4)**]. Please note the well-maintained lacuno-canalicular networks **(D2–D4)** between osteocytes and bone graft (turquoise *). Similarly, in the ScA and ScRA groups, good integration in connective tissue **(D3)** as well as direct attachment of osteocytes and newly formed bone tissue **(D4)** was observed [white * in **(C4)**]. Cellular extensions between the osteocytes [turquoise ** in **(D2)**] further stress the occurrence of well-orchestrated bone remodeling. BC, bone chip; CT, connective tissue; MU, muscle tissue; OT, osteocyte; SC, scaffold. Scale bars: **(A1–A4)**, 2,000 μm; **(B1–B4)**, 200 μm; **(C1–C4)**, 50 μm (except C4, 100 μm); **(D1–D4)**, 5 μm (except D4, 10 μm).

Further corroborating the results of the decalcified samples, all experimental groups showed excellent integration into the surrounding host tissue in undecalcified samples (Figures 7A1–A4). The Voronoi mPCL-HA scaffolds alone (Sc group) showed excellent integration within the host tissue at 8 weeks post-implantation, particularly at the interface between the scaffold struts and connective tissue (Figure 7B1). Similarly, the treatment groups that included bone chips (the ScRIA2 and ScRA groups) showed good acceptance of the graft material and integration of the scaffold within the surrounding host tissue (Figures 7B2, B4). Notably, excessive soft tissue encroachment was avoided, and a small muscle layer derived from the rat’s panniculus carnosus integrated well with the outer surface of the scaffold wall was observed (Figure 7B3). Scanning electron microscopy imaging of the bone chips showed a viable osteocyte network within the graft fragments (see Supplementary Figure S12). Neovascularization with new blood vessels sprouting was observed (Figure 7C2—white circle dashed lines) throughout the bone graft and within the newly formed tissue surrounding the scaffold struts. Figures 7C2, C4 show white triangles pointing toward areas of bone remodeling, with osteoblastic osteocytes aligned at the interface of bone graft remnants and new bone formed (white asterisks in Figure 7C4, indicating well-integrated bone chip remnants). Similarly, blood vessels (white circle dashed lines) were also identified at the interface between the bone graft fragments and the new tissue being formed (Figure 7C2—osteocytes indicated with white asterisks in 7C2). The scaffold struts appeared to guide the collagen fiber orientation and alignment (Figures 7B3, C3, D3). Notably, crosstalk via lacuno-canalicular networks was observed between the osteocytes and bone chips and also the newly formed (bone) tissue (Figures 7D2, D4, see also Supplementary Figure S12).

## 4 Discussion

Over the past four decades, a steadily increasing number of studies has been published on the design and manufacture of scaffolds for bone tissue engineering (Supplementary Figure S13) (Hutmacher et al., 2023). Yet, only a small number of scaffolds have been translated from bench to bedside (Hollister, 2009; Hollister and Murphy, 2011; Hutmacher et al., 2023). Generation 1.0 and 2.0 scaffolds have been used clinically for more than 10 years after rigorous preclinical testing (Kobbe et al., 2020; Laubach et al., 2022b; Castrisos et al., 2022). Generation 3.0, which is a calcium phosphate-coated version of Generation 2.0, underwent rigorous preclinical testing and will be used in clinical trials in the near future (Laubach et al., 2022a). Yet, Generation 1.0–3.0 scaffolds use rectilinear layering designs because certified melt-based 3D printers and additive manufacturing platforms in the past did not allow the fabrication of complex scaffold geometries using medical-grade biomaterials without the use of additive support materials and extensive post-processing (Qi et al., 2021). The requirements for the support material and post-processing present considerable complexities, not only in manufacturing, but also in regulatory approval processes (Placone et al., 2020; Dizon et al., 2021). With a newly developed additive manufacturing platform, complex biomimetic architectures such as the established Voronoi design can be printed using a medical-grade composite material that contains an osteoinductive bioactive agent, namely mPCL-HA (Zhou et al., 2020; Hutmacher et al., 2023). Therefore, we present the first study to characterize the physical properties and *in vivo* biocompatibility of this Generation 4.0 scaffold in an animal model in combination with xenogeneic bone graft loading.

In the present study, high-resolution µCT analysis confirmed that a state-of-the-art printing quality was achieved with sub-micron powder particles well mixed with mPCL. However, HA has a higher density than PCL; thus, during the 3D printing extrusion process it may not be evenly distributed in the PCL matrix and therefore more prone to aggregate and settle at the edge of the filament (a phenomenon often referred to as “phase separation” or in the context of 3D printing as “sedimentation”) (Ghilan et al., 2020; van der Heide et al., 2022). It is conceivable that the seemingly decentralized distribution of HA particles observed in the filament periphery could be beneficial for *in vivo* biocompatibility of the biodegradable scaffolds, thereby further improving the biocompatibility and osteoconductivity and potentially the osteoinductivity of the composite scaffold (Lin et al., 2021). Importantly, at the scaffold nodes, excellent union and integration were achieved between struts. This feature has been shown to be crucial for efficient transmission of mechanical forces, even if thinning of the strut thickness might be observed during surface degradation (Lam et al., 2007). The mechanical properties of the Voronoi scaffolds fit well within the range of scaffolds previously used successfully in large animal models, with ≥70% porosity and fully interconnected pores printed with rectilinear filling and associated Young’s modulus of 22.2 MPa (Reichert et al., 2012; Sparks et al., 2020). The observed marginally higher actual porosity in the mPCL-HA Voronoi scaffolds compared to the design is not unexpected and could be due to a slight shrinkage of the PCL during the post-extrusion cooling process or influenced by process parameters such as printing speed, layer thickness and extrusion temperature (Jia et al., 2023). However, in particular, since a higher porosity of >70% was determined with simultaneous proper mechanical stability, suitability for *in vivo* testing could be ascertained. The minimal degradation observed here (less than 1% over 6 months) was optimal, which is a key requirement in SGBR to provide a temporary architecture to facilitate bone restitution throughout the entire process of bone regeneration (Fu et al., 2021). No pronounced autocatalysis was observed, in line with previous studies, which indicates a suitable *in vitro* degradation profile for the tested mPCL-HA Voronoi scaffolds (Hou et al., 2022), with only minor changes in mass, M_w_, and crystallinity. As crystallinity variations affect the mechanical properties of scaffolds (Hou et al., 2022), the maintenance of crystallinity of the mPCL-HA Voronoi scaffolds within the timeframe studied implies that the mechanical properties will be driven by new bone formation and remodeling only, as initiated by the regeneration process and not dictated by the degrading scaffold in the initial critical stages of bone regeneration.

Despite the moderate Mw change, no loss of scaffold integrity was observed as the mass loss was <1% over a period of 6 months. Thus, the findings of high porosity, suitable mechanical properties, and slow degradation profile, which matched the basic requirements for successful translation to the SGBR concept, prompted the implantation of scaffolds in partially immunodeficient rats to assess their biocompatibility and interplay with different clinically-relevant bone graft materials. Although scaffold designs have been continuously optimized throughout the last decade, sufficient bone healing has only been achieved when biological components such as bone grafts are added (Reichert et al., 2012; Tetsworth et al., 2019; Henkel et al., 2021; Laubach et al., 2022b). As such, equipping scaffolds with osteogenic cells alone did not achieve sufficient bone regeneration (Reichert et al., 2012), as extracellular matrix (ECM) components are also required (Berner et al., 2015). This finding, in turn, conclusively indicates that loading scaffolds with bone graft is the most feasible alternative for achieving bone regeneration in large volume bone defects (Berner et al., 2015; Tetsworth et al., 2019; Kobbe et al., 2020; Laubach et al., 2022b). Our results showed that the different bone graft types used in the clinic, in combination with the mPCL-HA Voronoi scaffold, have good biocompatibility *in vivo*, confirming previous literature that tissue-engineered constructs of biomaterial-RIA 2 bone graft (Laubach et al., 2023b), biomaterial-bone marrow (Moro-Barrero et al., 2007; Bansal et al., 2009) and biomaterial-bone chips (Laubach et al., 2022b) are valuable options to achieve bone regeneration. Specifically—although this was not the hypothesis of this work—we showed that utilizing bone chips, bone marrow, or a combination of both was associated with superior bone volume compared to bone marrow alone. This is in line with previous literature (Laubach et al., 2023a) where the bone matrix is host to various growth factors such as insulin-like growth factor 1 (Seck et al., 1998), bone morphogenetic protein (BMP)-2 and BMP-4 (Sipe et al., 2004; Dimitriou et al., 2005; Pecina and Vukicevic, 2007), and transforming growth factor-ß1 (Perez et al., 2018) that are deeply embedded in the bone matrix and thus sustainably released over time as the matrix is degraded and remodeled (Canalis et al., 1988; Joyce et al., 1990). Such controlled release of growth factors is not possible with bone marrow, which is rapidly resorbed by the body, thus limiting sustained bone regeneration. In any case, the feasibility of loading the mPCL-HA Voronoi scaffolds with different types of bone graft has been demonstrated. Importantly, in support of the clinically-relevant SGBR concept, haptic feedback from the surgeons indicated no marked differences in scaffold loadability between groups, as pores could be filled with bone graft with ease and homogeneity, as confirmed by μCT-scanning.

Scaffolds fabricated from mPCL alone are known to promote an innate immune response (Baker et al., 2011); however, the subcutaneous implantation of the mPCL-HA composite scaffold resulted—as expected from clinical studies (Laubach et al., 2023b; Liodakis et al., 2023)—in a minimal non-inflammatory response as depicted by the IHC results for all experimental groups. In particular, we observed weak staining of foreign body giant cells with iNOS (M1 macrophage phenotype marker), indicating a balanced host defense to the scaffold-bone graft constructs. Excessive M1 polarization due to an extensive inflammatory foreign body reaction would have led to fibrous encapsulation and lack of extensive neovascularization and, ultimately, failure of the mPCL-HA scaffold integration (Graney et al., 2016). Interestingly, we did see endochondral bone formation on the scaffold-bone graft interfaces. It has been suggested that osteoblast differentiation can be induced by alternatively activated macrophages, including osteoclasts (Gu et al., 2017), which in turn can overturn pro-inflammatory responses and scavenge the by-products of biomaterials. As the inflammatory response elicited by implanted biomaterials is particularly modulated by characteristics such as scaffold morphology that can alter macrophage functions such as survival, adhesion, and secretion (Yim and Leong, 2005; Tan et al., 2006; Ainslie et al., 2009), the novel mPCL-HA Voronoi scaffold design used facilitated *in vivo* tissue integration. In line with literature (Lam et al., 2009), internal autocatalysis was neither observed for scaffolds *in vivo*, as intact cross-sections of scaffold struts with no “hollowed-out” structures were detected during the undecalcified sample examinations. Despite containing substantial supply of bone growth factors, bone grafts, when implanted in bone defects without scaffold support, are often rapidly resorbed due to the particle size and the absence of native bone matrix and also due to the inherently faster soft tissue formation compared to bone regeneration (Laubach et al., 2023b). The histological results observed in the present study suggest that the interconnected porous architecture of the mPCL-HA scaffold served as an osteoconductive matrix by providing the structural basis (Giannoni et al., 2008) for successful load of bone graft, initial deposition of structured soft tissue, and neovascularization. The beneficial biological environment observed in this study is in line with previous findings of enhanced neovascularization facilitated by the >70% porosity of the scaffold eliciting diffusion of oxygen and nutrient supply over short distances (Giannoni et al., 2008).

Furthermore, current mechanobiology theories indicate that osseous interfragmentary strain, stress, and fluid flow influence cell differentiation (Epari et al., 2010). Therefore, a reasonable hypothesis to explain the beneficial (bone) tissue growth observed in SGBR might be that entrapping bone graft within the scaffold interconnected pores reduces the interfragmentary strain. The reduction in interfragmentary strain between the small bone chips might induce and facilitate bone regeneration (Claes et al., 1997; Claes et al., 1998; Claes et al., 2018), as evidenced by our histological and SEM results. The small bone chip fragments served as osteoinductive surfaces and also provided osteoconductive attachment sites for bone-forming cells, thereby supporting and guiding bone formation directly onto their surfaces (Franchi et al., 2004). For illustrative purposes, SEM images derived from a different study showing dead osteocytes are depicted in Supplementary Figure S14. Literature indicates that during the surgical bone graft-harvesting processes, the lacuno-canalicular networks associated with the osteocytes nearest to the fragment surface might be disrupted (Shah and Palmquist, 2017). In the current study, we observed that the re-implanted ovine bone graft osteocytes remained viable and functional. Thus, in line with previous studies (Roberts and Rosenbaum, 2012; Shah and Palmquist, 2017), viable and functional re-implanted bone graft was associated with slow resorption of graft fragments with simultaneous deposition of new bone at the periphery of the graft eventually resulting in successful bone regeneration throughout the mPCL-HA Voronoi scaffolds loaded with bone graft.

### 4.1 Limitations

*In vitro* degradation analyses, even under the closest possible approximation to physiological conditions, such as those used in this work, are limited in their capacity for translation to living organisms. Polycaprolactone is biodegradable but only slowly hydrolytically bioresorbable *in vivo* (Vert, 2007; Lam et al., 2009). Therefore, the long-term fate of by-products of the novel mPCL-HA Voronoi scaffolds may need to be elaborated in future long-term *in vivo* and subsequent clinical studies. In particular, the biomechanical properties of the novel Generation 4.0 mPCL-HA Voronoi scaffolds need to be investigated in future long-term *in vivo* studies and compared with those of Generation 1.0–3.0 scaffolds. Furthermore, considering a sufficiently balanced immune response from a functioning immune system is essential for the initiation of the bone healing cascade and also for an effective healing sequence (Park and Barbul, 2004; Serhan and Savill, 2005), bone regeneration may have been affected by the chosen animal model. Depending on the type of T cell [i.e., negative (e.g., regulatory T helper cells) or positive (e.g., CD 8^+^ T cells)], regenerative effects on (bone) healing have been observed (Reinke et al., 2013; Schmidt-Bleek et al., 2015; Kyriakides et al., 2022). For instance, for allogeneic acellular skeletal muscle grafts implanted in immunodeficient (RNU, Foxn1-deficient) and immunocompetent Sprague Dawley rats, differences in muscle regeneration were observed (McClure et al., 2021). To avoid rejection of fresh bone graft, partially immunodeficient rats were used in the current study. Furthermore, to increase external validity and, in particular, to account for the osteoimmunomodulatory effect of the animal model and thus improve overall translatability, we used outbred animals that were not fully immunodeficient (Dirnagl et al., 2022). Nonetheless, as the interaction with the immune system is key to analyzing the SBGR concept, implantation in an orthotopic location and the use of large fully immunocompetent animals are suggested for future studies to validate the regenerative potential shown here.

## 5 Conclusion

In this study, we observed that the 3D-printed mPCL-HA Voronoi scaffolds possess the previously described key properties of SGBR: high porosity of >70%, suitable scaffold morphology with fully interconnected strut architecture, and *in vivo* biocompatibility, all of which are essential for promoting cell attachment, migration, proliferation, and differentiation and are thus critical for guiding bone regeneration. This study supports the use of mPCL-HA Voronoi scaffolds to successfully advance the SGBR concept toward future large preclinical animal studies and ultimately in clinical trials.

## Data Availability

The raw data supporting the conclusions of this article will be made available by the authors, without undue reservation.
