# Supplementary material for: In vivo characterization of 3D-printed polycaprolactone-hydroxyapatite scaffolds with Voronoi design to advance the concept of scaffold-guided bone regeneration
Source: Front Bioeng Biotechnol. 2023 Oct 4;11:1272348. doi: 10.3389/fbioe.2023.1272348 (PMC10584154; doi:10.3389/fbioe.2023.1272348)
Supplement: Supplementary file 5 [file DataSheet1.docx]

Supplement

Contents

[Supplementary table - 3 -](#_Toc145684888)

[Supplementary Table 1. Primary antibodies specific to the osteogenic, macrophages and vascularization markers used for this study, as well as protocol specifications. - 3 -](#_Toc145684889)

[Supplementary figures - 5 -](#_Toc145684890)

[Supplementary Figure 1. Scanning electron microscope (SEM) images depicting the scaffold generations (1.0 - 4.0) designed and fabricated for the concept of scaffold-guided bone regeneration. - 5 -](#_Toc145684891)

[Supplementary Figure 2. Stereolithography (STL) file of the Voronoi scaffold design. - 6 -](#_Toc145684892)

[Supplementary Figure 3. Setup of the mechanical compression testing of the mPCL-HA Voronoi scaffolds conducted under simulated physiological conditions. - 6 -](#_Toc145684893)

[Supplementary Figure 4. Clinically relevant intramedullary harvesting methods for obtaining bone grafts from the sheep femur. Please note the R-A method was applied following removal of bone marrow. R-A method, reaming-aspiration method; RIA 2 system, Reamer-Irrigator-Aspirator 2 system. Adapted from Ref (Laubach et al., 2023). Partially created with BioRender.com. - 7 -](#_Toc145684894)

[Supplementary Figure 5. Biosafety cabinet setup for anesthesia and surgical procedures in partially immunocompromised (nude) rats. - 8 -](#_Toc145684895)

[Supplementary Figure 6. Loading of 3D-printed mPCL-HA Voronoi scaffolds with fresh ovine bone grafts and depiction of surgical procedures. - 9 -](#_Toc145684896)

[Supplementary Figure 7. Representative scaffold reconstruction used for porosity calculation with μCT data. - 10 -](#_Toc145684897)

[Supplementary Figure 8. Number-averaged molecular weight (Mn) of mPCL-HA Voronoi scaffolds over 180 days of degradation. - 11 -](#_Toc145684898)

[- 11 -](#_Toc145684899)

[Supplementary Figure 9. Dispersity index of mPCL-HA Voronoi scaffolds for assessing degradation over 180 days. - 11 -](#_Toc145684900)

[Supplementary Figure 10. Embedded osteon indicating new bone formation on the surface of original bone graft (ScRIA2 group). - 12 -](#_Toc145684901)

[Supplementary Figure 11. Osteocyte viability demonstrated in a scanning electron microscopy image and in images with immunohistochemical stains. Origin of the images according to the experimental groups: A, C, D-F, ScRIA2 group; B, ScRA group. - 13 -](#_Toc145684902)

[Supplementary Figure 12. Representative sequence of scanning electron microscopy images showing crosstalk via lacuno–canalicular networks observed between osteocytes with the bone chips, as well as with newly formed (bone) tissue (ScRA group). - 14 -](#_Toc145684903)

[Supplementary Figure 13. Annual growth of studies on scaffolds for bone tissue engineering published in PubMed between 1996 and 2022. - 15 -](#_Toc145684904)

[Supplementary Figure 14. Illustrative scanning electron microscopy images of bone with dead osteocytes derived from a separate study in which pigs were treated with bisphosphonates (unpublished original data). - 16 -](#_Toc145684905)

[References - 17 -](#_Toc145684906)

# Supplementary table

## Supplementary Table 1. Primary antibodies specific to the osteogenic, macrophages and vascularization markers used for this study, as well as protocol specifications.

| **Antibody** | **Specimen tissue site** | **Reactivity** | **Raised in** | **Clonality** | **Cat. no.** | **Antigen retrieval** | **Blocking** | **Dilution** | **Incu-bation** | **DAB** | **Description** |
| --- | --- | --- | --- | --- | --- | --- | --- | --- | --- | --- | --- |
| CD68  (Cluster of Differentiation 68) | Rat | Mouse, rat | Rabbit | Polyclonal | ab125212 | Proteinase K 5 min | 2% BSA 30 min | 1:300 | 1h | 25 s | M1 and M2 macrophage marker |
| iNOS  (nitric oxide synthase) | Rat | Mouse, rat | Rabbit | Polyclonal | ab15323 | Proteinase K 5 min | 2% BSA 30 min | 1:100 | 1h | 2:45 min | M1 macrophage, pro-inflammatory |
| MR  (mannose receptor) | Rat | Mouse, rat, human | Rabbit  Mouse | Polyclonal | ab64693 | Proteinase K 5 min | 2% BSA 30 min | 1:100 | 1h | 40 s | M2 macrophage, pro-regenerative |
| vWF  (von Willebrand factor) | Rat | Human | Rabbit | Polyclonal | IR527 | Proteinase K 5 min | 2% BSA 30min | Ready to use | 1h | 2:30 min | Mature blood vessel |
| COL I  (collagen type I) | Rat | Mouse, Rat, Sheep, Goat, Horse, Cow, Human, Pig | Rabbit | Monoclonal | ab138492 | Proteinase K 5 min | 2% BSA 30 min | 1:100 | 1h | 20 s | Early bone marker for osteoblastic differentiation |
| COL II  (collagen type II) | Rat | Human, Mouse, ovine | Mouse | Monoclonal | DSHB  II-II6B3 | Proteinase K 5 min | 2% BSA 30 min | 1:100 | 1h | 1 min | Endochondral bone formation at areas of cartilage matrix |
| OC  (osteocalcin) | Rat | Human | Rabbit | Monoclonal | ab 133612 | Proteinase K 5 min | 2% BSA 30 min | 1:100 | 1h | 30 s | Late osteogenic bone marker |

# Supplementary figures

##
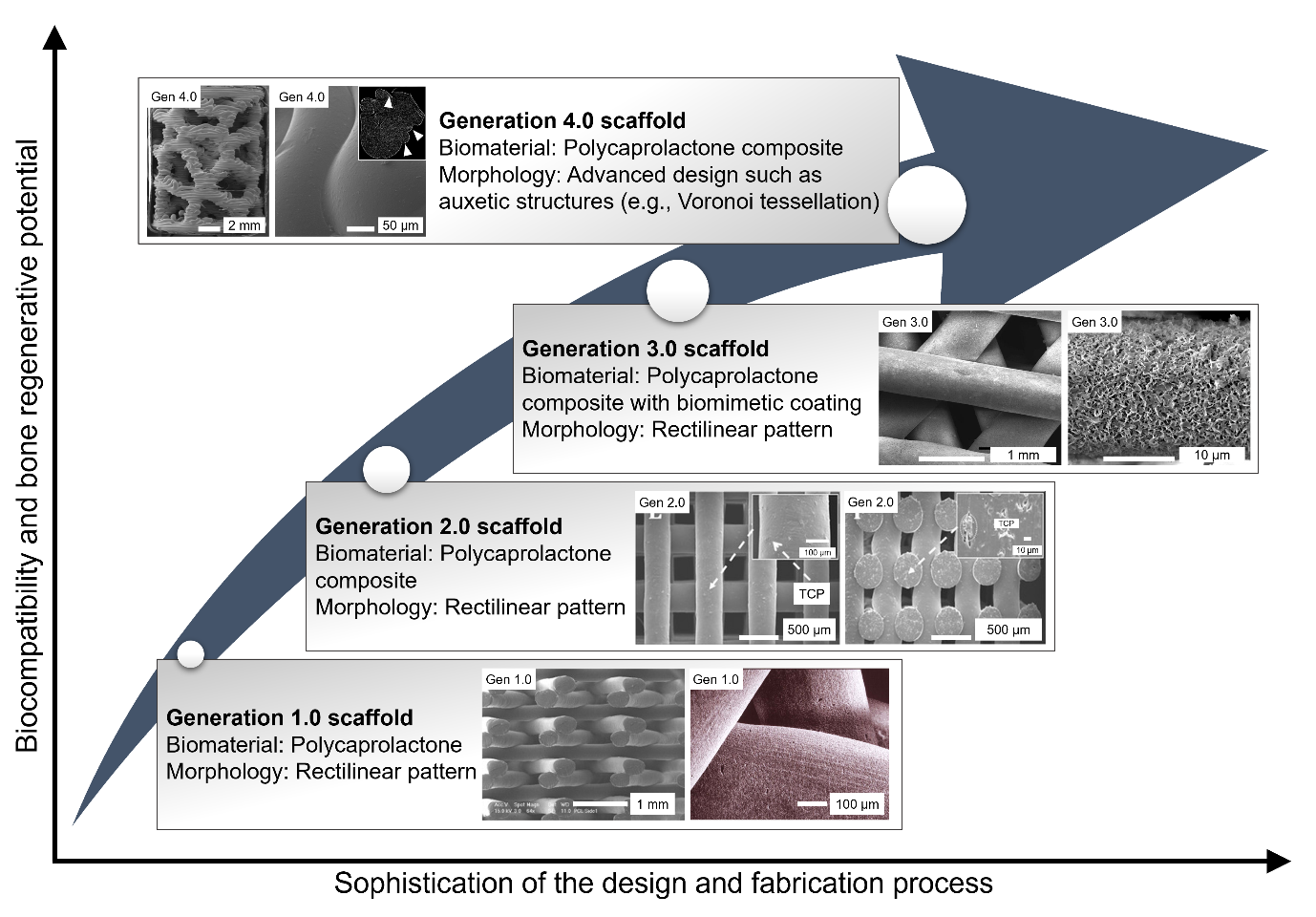
Supplementary Figure 1. Scanning electron microscope (SEM) images depicting the scaffold generations (1.0 - 4.0) designed and fabricated for the concept of scaffold-guided bone regeneration.

Generation 1.0 scaffolds are 3D-printed from polycaprolactone (PCL) using a rectilinear layering pattern (either alternating raster angles of 0°, 60° and 120°, or alternating raster angles of 0° and 90°) for each successive layer. Generation 2.0 scaffold are with rectilinear layering pattern using a PCL composite, such as the PCL-β-tricalcium phosphate (PCL-TCP, wt% 80:20) shown here: Top view and zoom-in view of the filament and scaffold cross-sectional view and zoom-in view of the cut surface. Generation 3.0 scaffolds are fabricated with a rectilinear layering pattern of a PCL composite and then subjected to an additional surface coating, such as the bioactive carbonated hydroxyapatite (CHA) coating of PCL-TCP scaffolds (left image) or calcium phosphate (CaP) coating (right image). Generation 4.0 scaffolds are made of a PCL composite, such as PCL-HA (wt% 96:4) shown here, 3D-printed with advanced designs such as the Voronoi tessellation. In this example, based on its identifiable filaments the 3D-printed Voronoi structure guarantees the largest possible surface to volume ratio to further increase the bone regenerative capacity of the scaffolds, whereby the HA particles are predominantly located at the filament edges (inset, white arrows) separated from the surface only by a very thin PCL layer. Left SEM image of Generation 1.0 scaffolds reproduced from Ref (Lam et al., 2009) with permission from John Wiley & Sons Inc. SEM images of Generation 2.0 scaffolds reprinted from Ref (Huang et al., 2018). Left SEM image of Generation 3.0 scaffolds reprinted from Ref (Arafat et al., 2011), with permission from Elsevier.

##
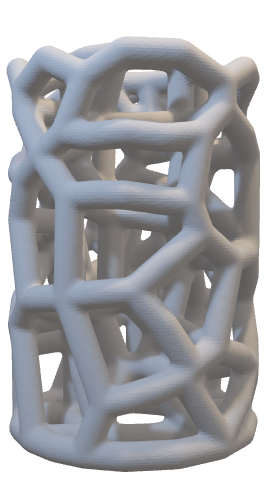
Supplementary Figure 2. Stereolithography (STL) file of the Voronoi scaffold design.


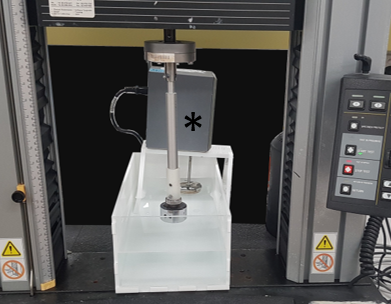


## Supplementary Figure 3. Setup of the mechanical compression testing of the mPCL-HA Voronoi scaffolds conducted under simulated physiological conditions.

To achieve the simulated physiological conditions at 37°C during compression testing, a custom-made instrument (*) for constant heating of the 1X phosphate buffered saline bath was used.

##
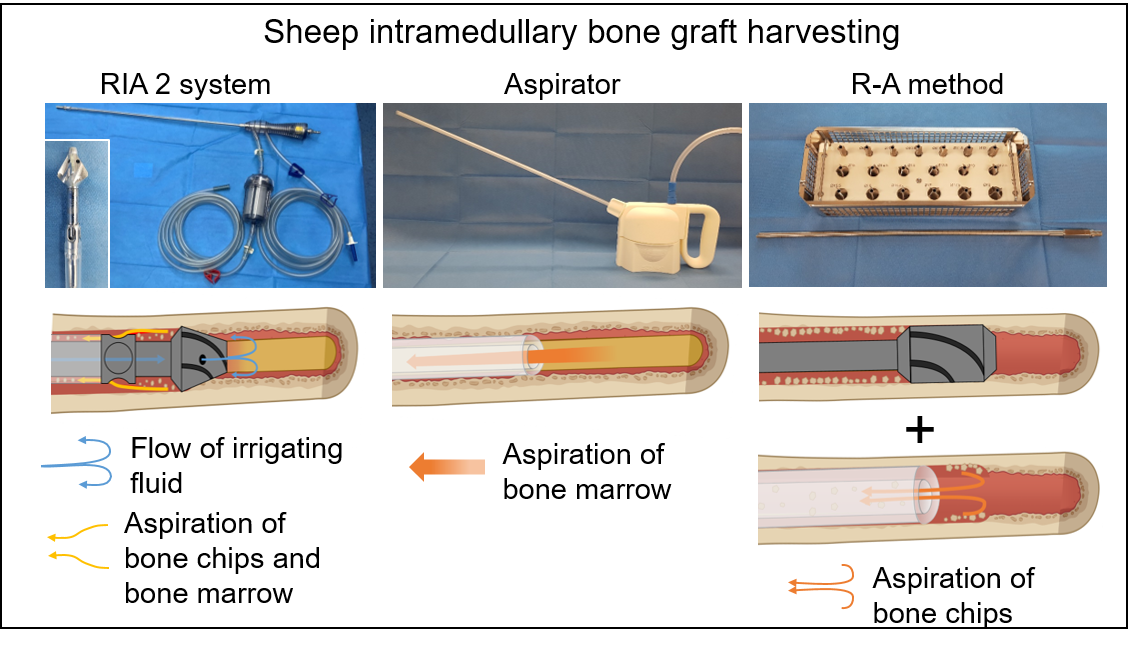
Supplementary Figure 4. Clinically relevant intramedullary harvesting methods for obtaining bone grafts from the sheep femur. Please note the R-A method was applied following removal of bone marrow. R-A method, reaming-aspiration method; RIA 2 system, Reamer-Irrigator-Aspirator 2 system. Adapted from Ref (Laubach et al., 2023). Partially created with BioRender.com.

##
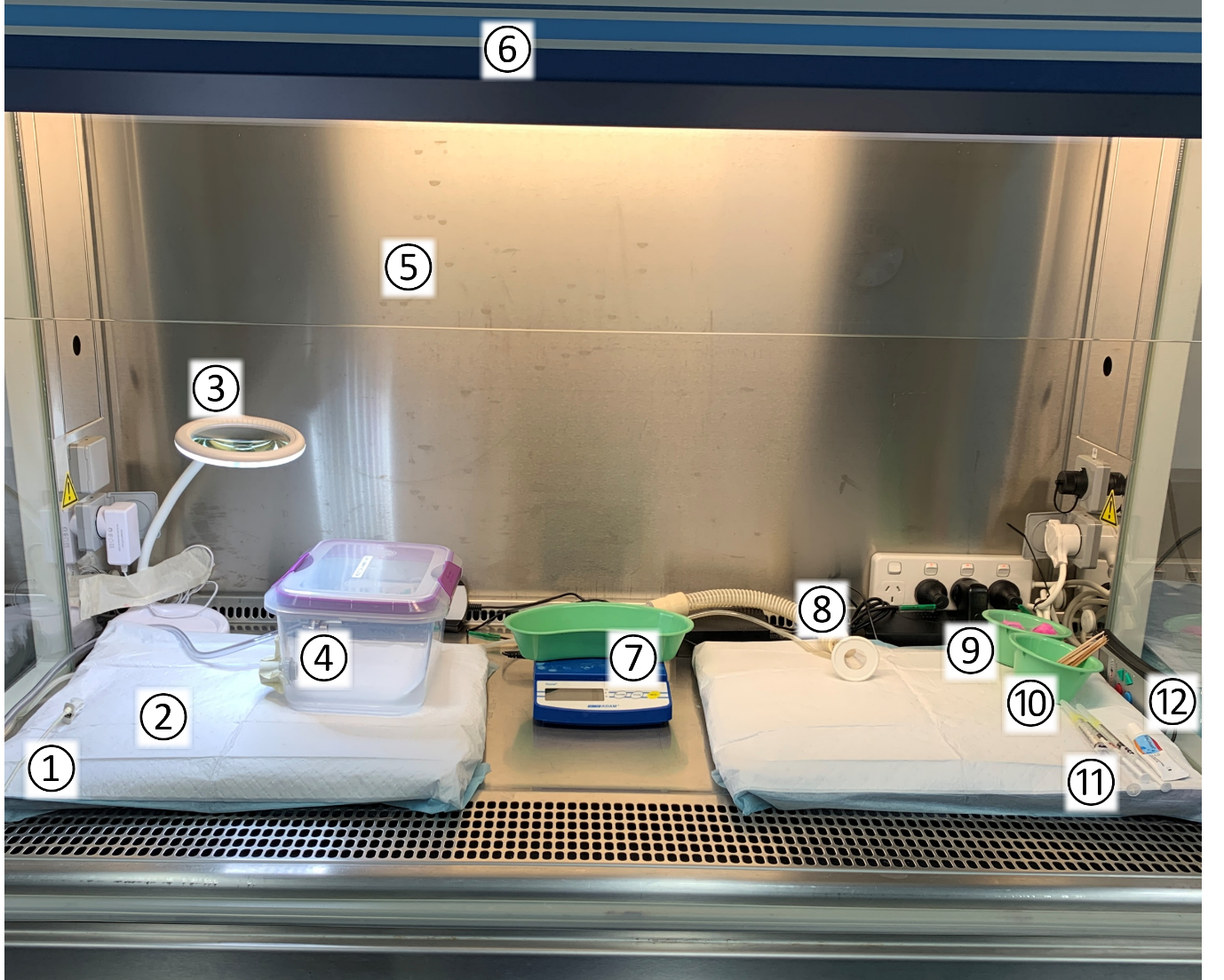
Supplementary Figure 5. Biosafety cabinet setup for anesthesia and surgical procedures in partially immunocompromised (nude) rats.

Key:

1 = pediatric pulse oximeter connected to patient monitoring station;

2 = covered heat mat pre-warmed to 37°C;

3 = lamp serving as an additional light source during surgery and as a heat source during animal recovery;

4 = anesthetic induction chamber with attached anesthetic gas inlet tube;

5 = sliding glass sash;

6 = Class II Biological Safety Cabinet;

7 = weigh scale with a sterilized kidney dish for containing the animals during weighing;

8 = funnel-shaped nose cone with a fitted flexible diaphragm connected to the anesthetic gas inlet and a waste anesthetic scavenge tube (breathing circuit);

9 = sterile pudding sponges in surgical antiseptic solution;

10 = Gallipot (surgical pot) with 80% (v/v) ethanol and cotton tips for disinfection of injection sites;

11 = syringes with needles for injections of pre-emptive analgesia, prophylactic antibiotic, and saline (replacement fluid);

12 = eye lubricant

##
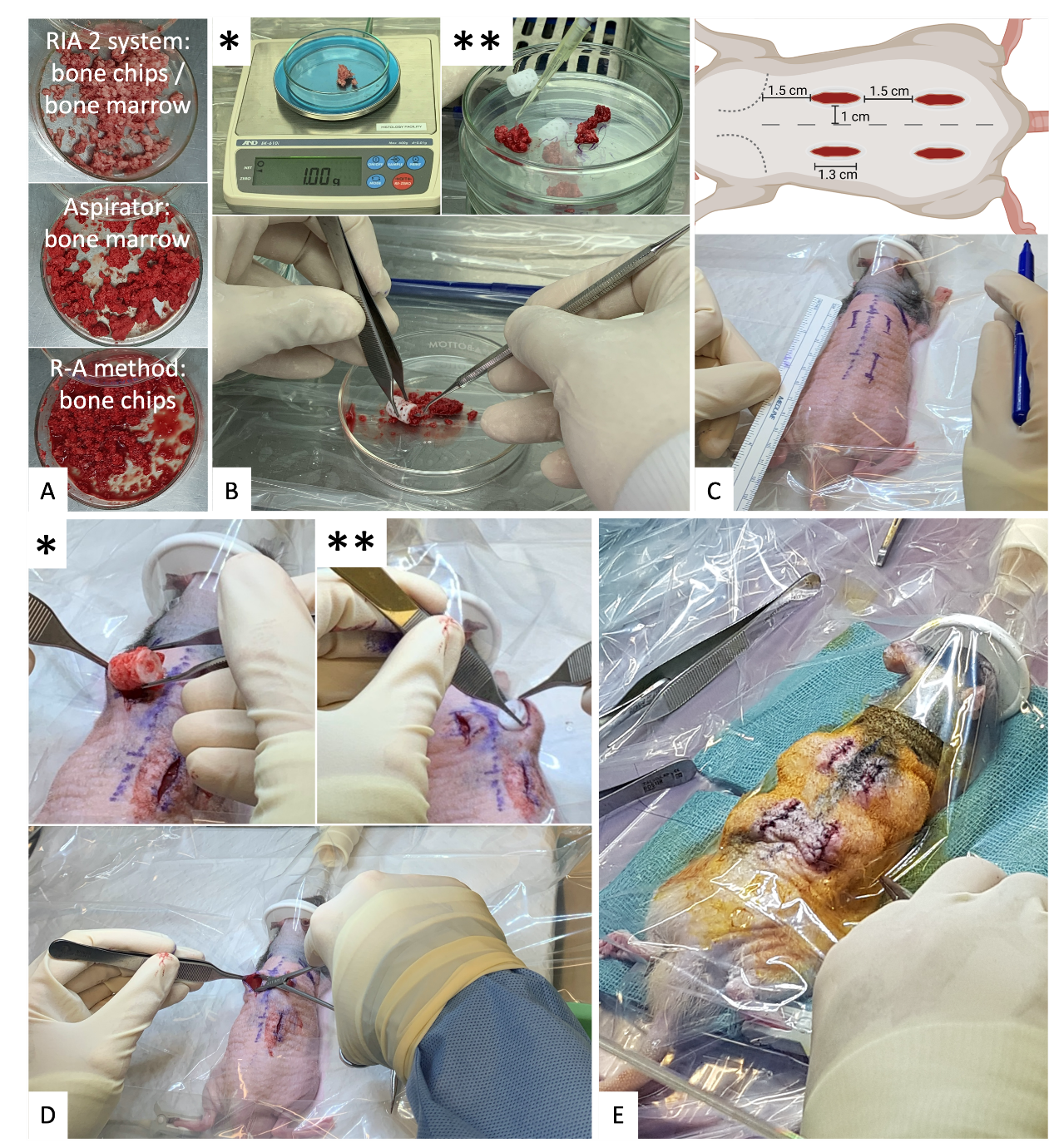
Supplementary Figure 6. Loading of 3D-printed mPCL-HA Voronoi scaffolds with fresh ovine bone grafts and depiction of surgical procedures.

The fresh ovine bone graft (A) is weighed in a sterile petri dish (* inset in B). The scaffolds and fibrin glue are added (** inset in B) prior to homogenously loading the bone graft using surgical instruments (B). Back area of fully anesthetized rat was prepared surgically and covered with a sterile plastic drape. Four skin incisions were made with sufficient distance from each other to avoid bone graft cross-contamination (C), as schematically highlighted by the landmarks in the inset. Subcutaneous pockets were then created with blunt dissection below the panniculus carnosus at each surgical site (D). Either a loaded (*inset in D) or a pristine (** inset in D) scaffold (scaffold alone) was placed horizontally in each subcutaneous pocket, with each animal receiving four constructs from different experimental groups. The incision was closed in layers with surgical sutures in a subcuticular pattern and the wounds were covered with a thin layer of wound aerosol topical spray (E). The animals were then allowed to recover from anaesthesia. Figure partially created with BioRender.com. R-A method, reaming-aspiration method; RIA 2 system, Reamer-Irrigator-Aspirator 2 system.

##
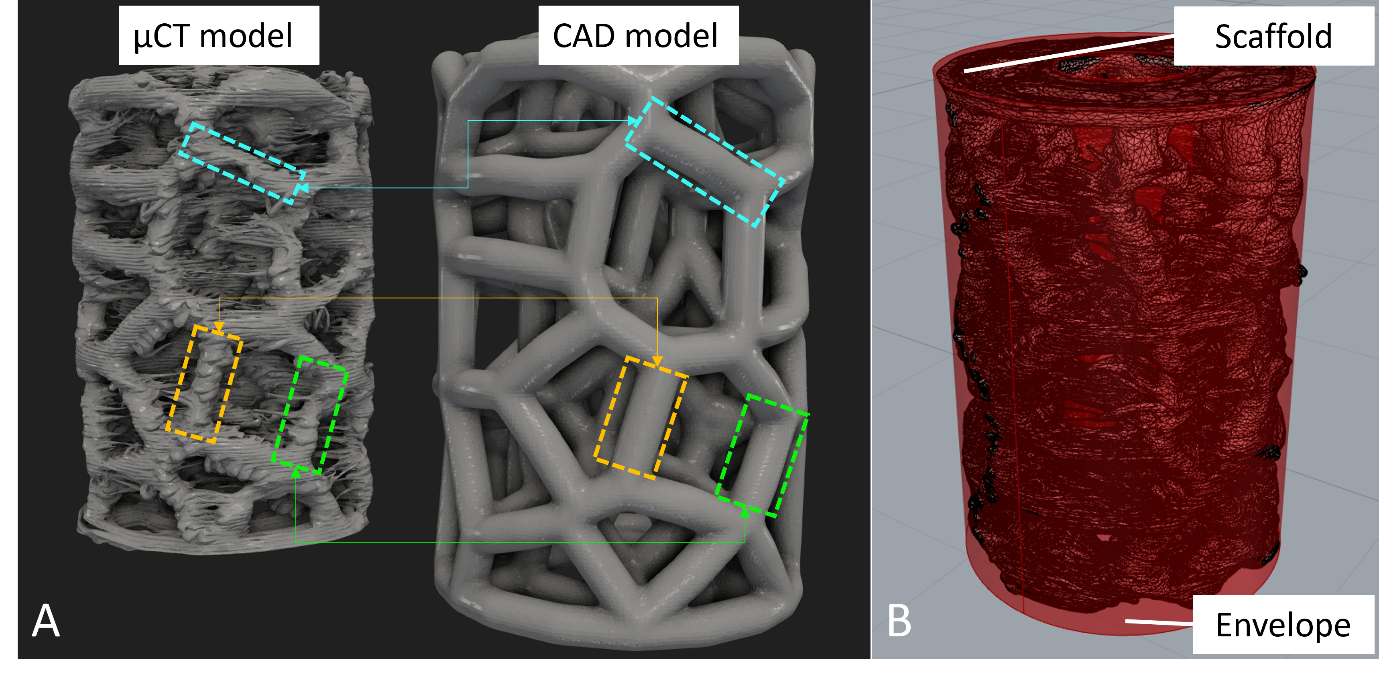
Supplementary Figure 7. Representative scaffold reconstruction used for porosity calculation with μCT data.

Due to thinner struts in the µCT model, a mean porosity of 72.8% is observed compared to the computer aided design (CAD) model porosity of 63.3% (A). Representative depiction showing the calculation of the porosity of the µCT model in which in an envelope was sculpted around the scaffold and the scaffold volume subsequently subtracted from the volume filling the envelope (B).

##
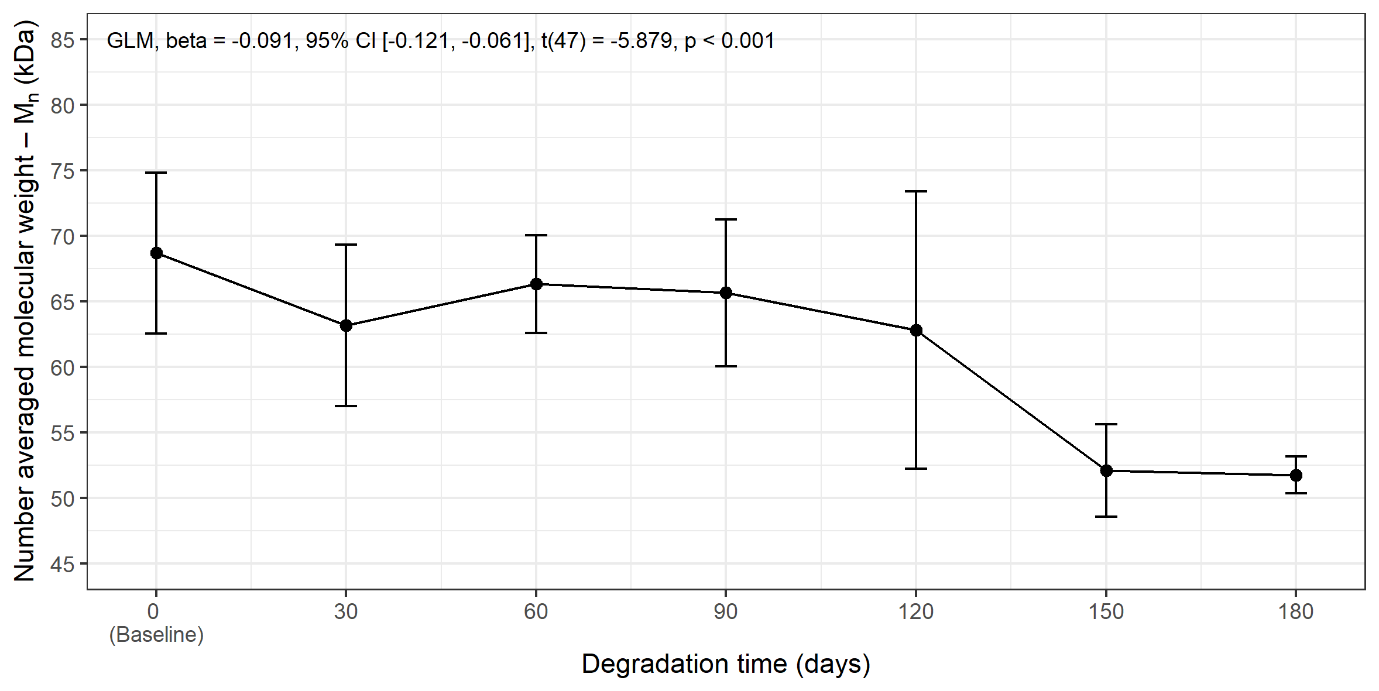
Supplementary Figure 8. Number-averaged molecular weight (Mn) of mPCL-HA Voronoi scaffolds over 180 days of degradation.

The effect of the timepoint is statistically significant and negative, and a reduction of M_n_ was observed, particularly between baseline and day 30, as well as between day 120 and 150. However, overall, the reduction in M_n_ over 180 days was small.

##
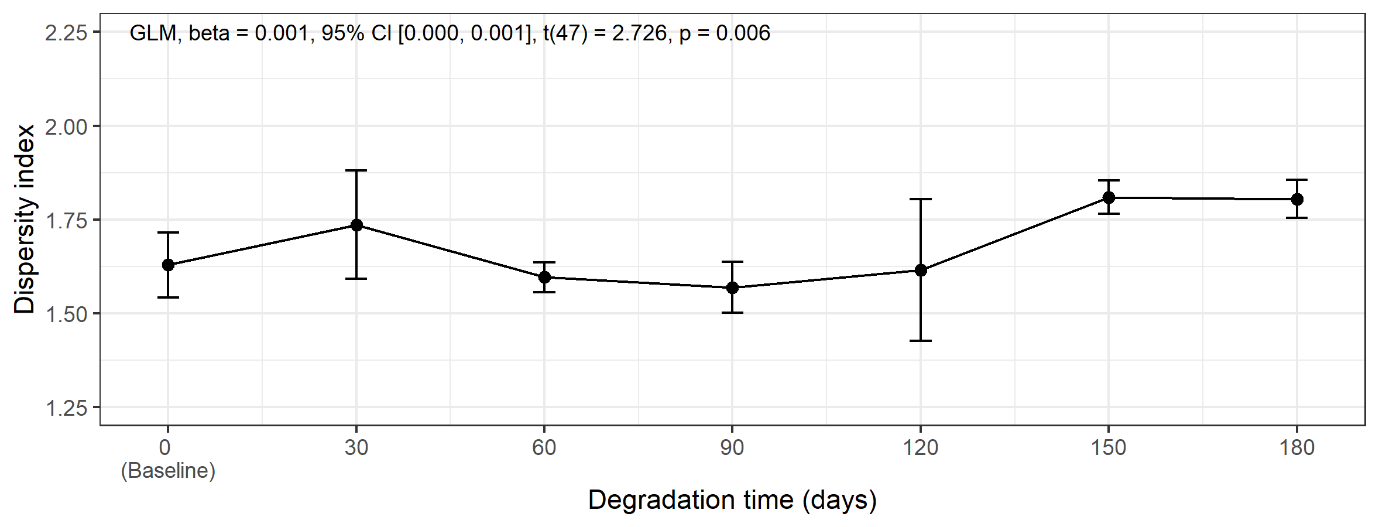


## Supplementary Figure 9. Dispersity index of mPCL-HA Voronoi scaffolds for assessing degradation over 180 days.

The effect of the timepoint is statistically significant and positive; however, very little difference was observed in the dispersity index during the follow-up period.

##
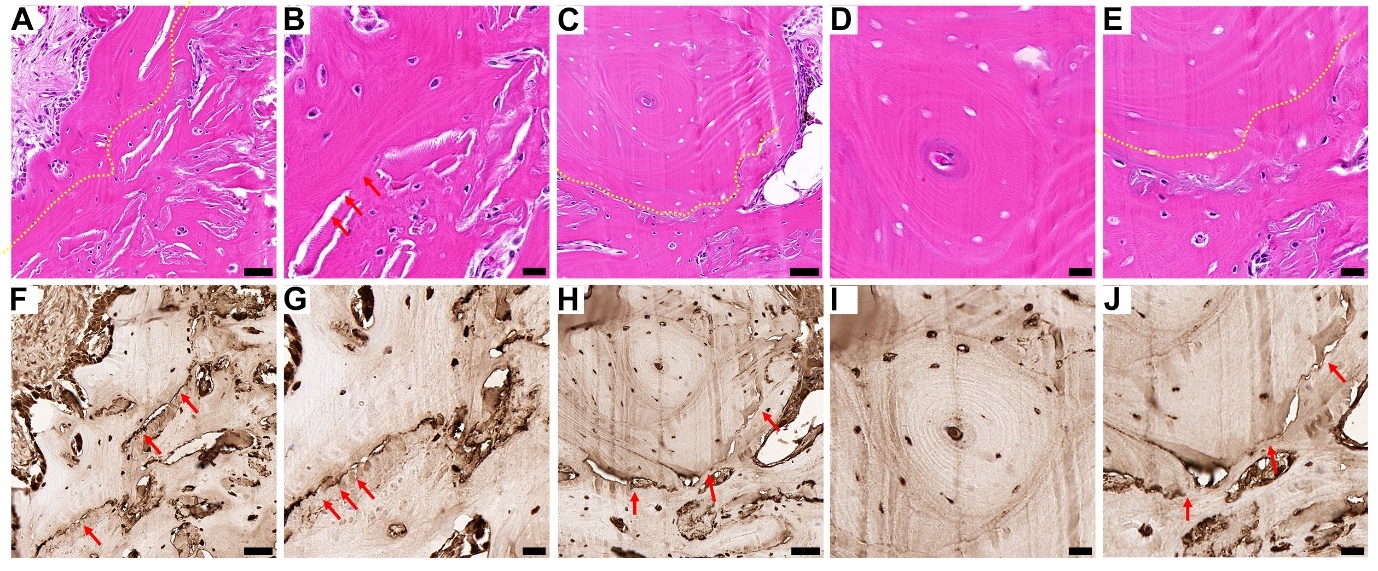
Supplementary Figure 10. Embedded osteon indicating new bone formation on the surface of original bone graft (ScRIA2 group).

Tiny fragments of the original bone graft (yellow arrows) (A, B), as well as an osteon (C-E) embedded within the new forming bone tissue. The yellow dotted line in C and E are demarking the interface of the original bone graft and newly formed bone. The interface of the original bone graft fragments and the newly forming bone is also clearly demarcated and depicted by osteocalcin staining (F – J, red arrows), which was strongly expressed at osteocytes and osteoblast cells. Scale bars: A, C, D, F, 50 µm; B, D, E, G, J, 20 µm.

##
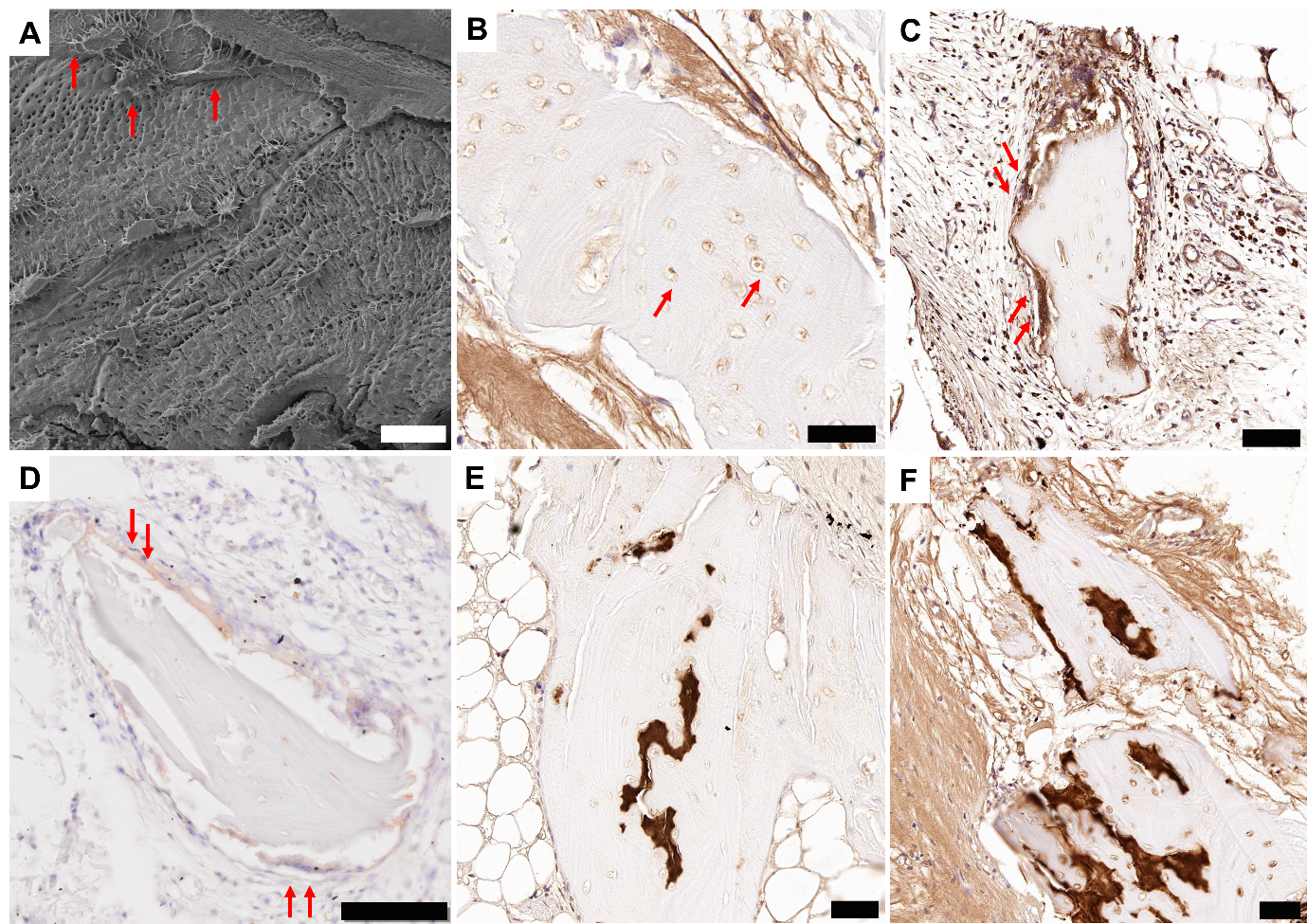
Supplementary Figure 11. Osteocyte viability demonstrated in a scanning electron microscopy image and in images with immunohistochemical stains. Origin of the images according to the experimental groups: A, C, D-F, ScRIA2 group; B, ScRA group.

Scanning electron microscopy of the graft fragment showing viable osteocytes (A) as depicted in direct contact with osteoblastic-osteocyte cells on the surface of the bone graft fragment (red arrows in A) Moreover, osteoprotegerin immunohistochemical staining (B) illustrates viable osteocytes residing bone graft fragments (red arrows in B). Osteoclast activity (C) and osteoblast recruitment at tiny bone graft fragments (D, red arrows), depicting bone remodelling process. Newly formed cartilage is also observed through collagen type II expression within the bone graft fragment (E, F). Part of the ossicles undergo remodelling via osteochondral bone formation in the center of the bone graft fragment (at the haversian canals), however indirect bone formation is also seen on the outer surface of the graft (E, F). Immunohistochemical stains: B, osteoprotegerin; C, CD68; D, alkaline phosphatase; E, F, collagen type II. Scale bars: A, 20 µm; B, E, F, 50 µm; C, D, 100 µm.

##
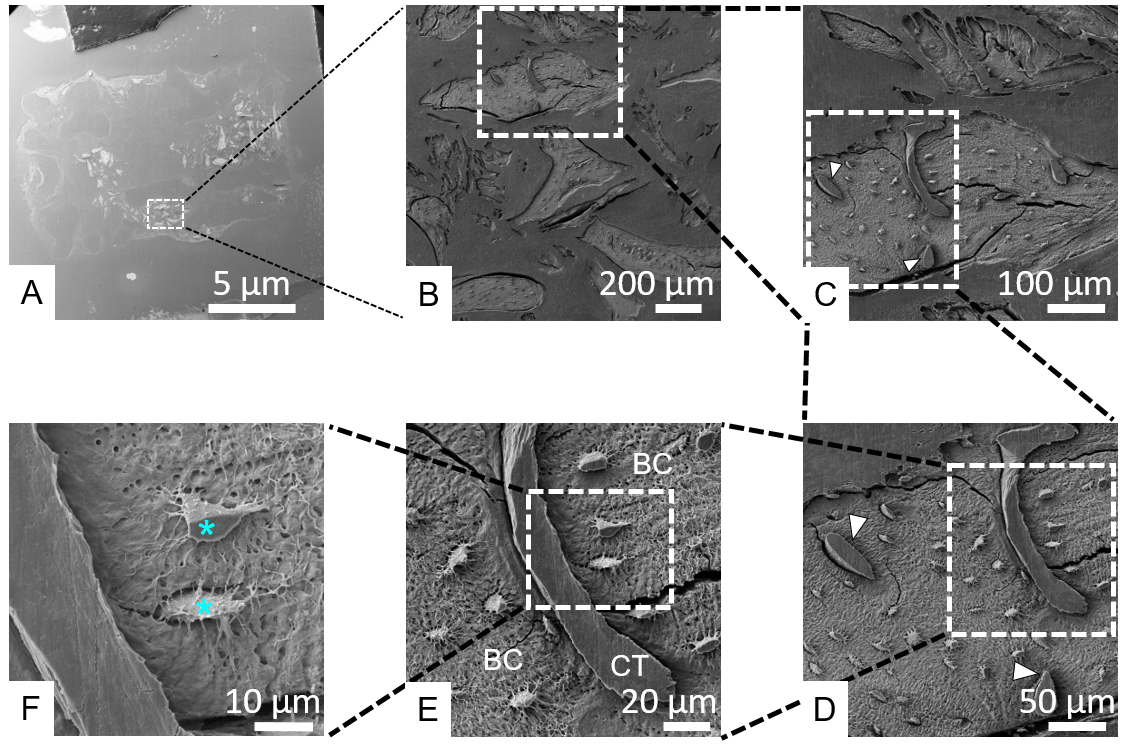
Supplementary Figure 12. Representative sequence of scanning electron microscopy images showing crosstalk via lacuno–canalicular networks observed between osteocytes with the bone chips, as well as with newly formed (bone) tissue (ScRA group).

The white dashed rectangle in each image shows the corresponding section used for magnification in the following image, using a sample from ScRA group as an example in this figure (A). Well-integrated bone fragments are evident in the connective tissue (B) including a multitude of osteocytes covering the bone fragments (C). The viability of the osteocytes might be in particular associated with rich vascularization; triangles point toward bone chips piercing blood vessels (C and D). Viable osteocytes are close to connective tissue, with cross-talk via lacuno-canalicular networks to bone chips (E) and between osteocytes (*) and bone chips (F). BC, bone chip; CT, connective tissue.

##
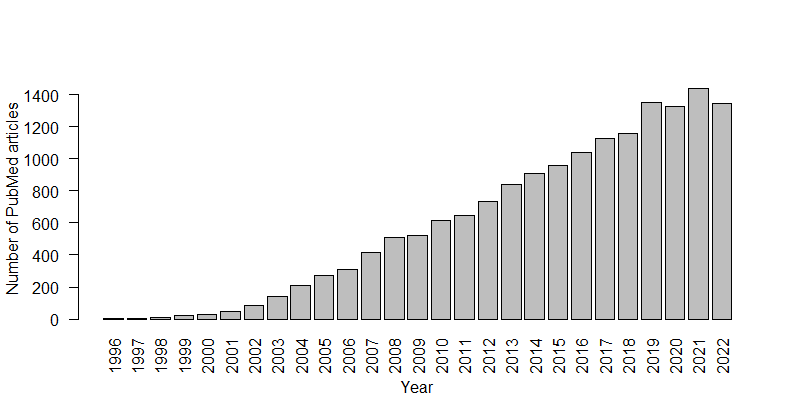
Supplementary Figure 13. Annual growth of studies on scaffolds for bone tissue engineering published in PubMed between 1996 and 2022.

The search and bar chart illustrations were performed on 05.12.2022 using the RISmed package in R statistical software (version 4.0.2; R Foundation for Statistical Computing, Vienna, Austria) and had the following search strategy: ((engineering) AND (bone)) AND (scaffold).

##
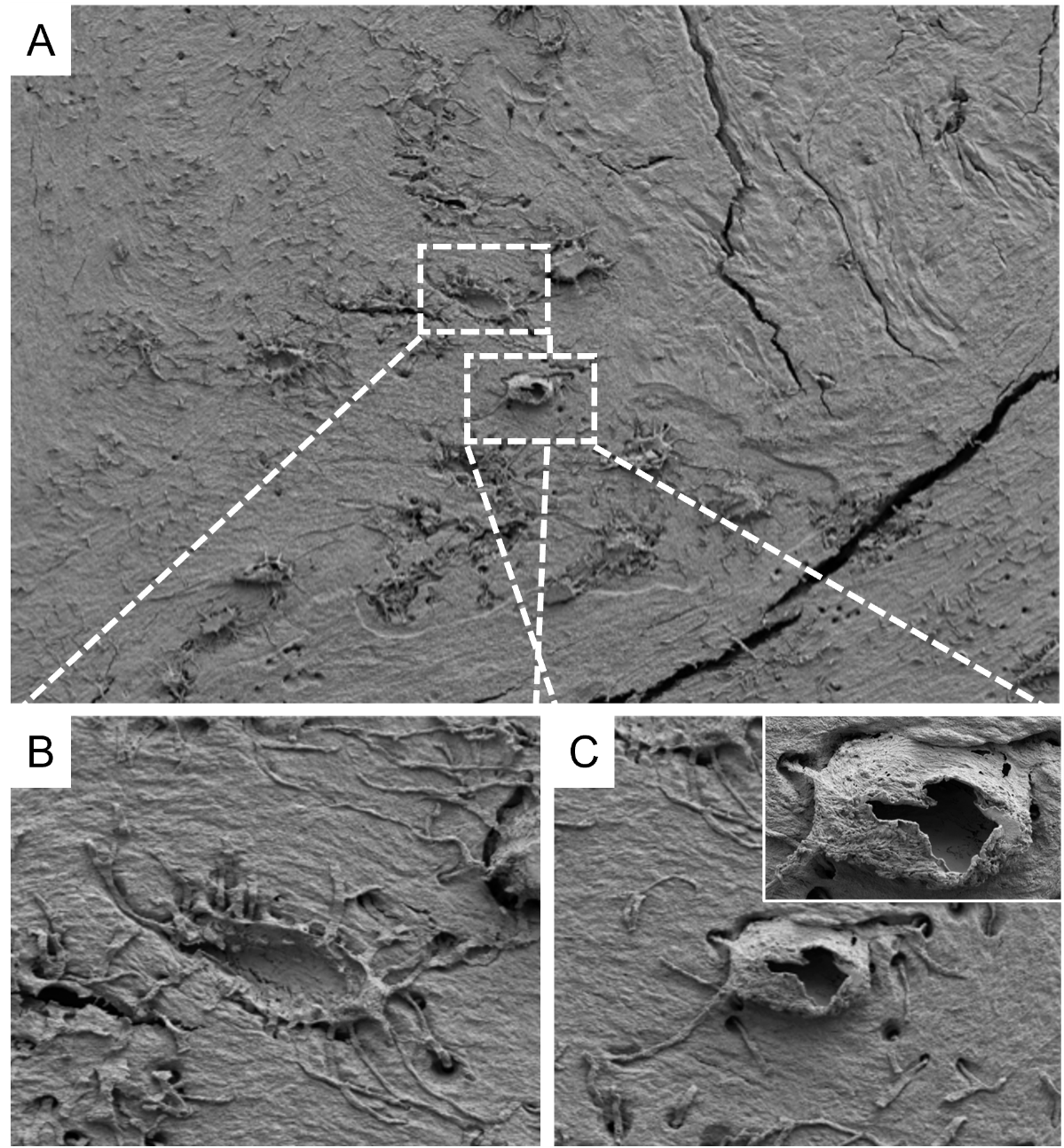
Supplementary Figure 14. Illustrative scanning electron microscopy images of bone with dead osteocytes derived from a separate study in which pigs were treated with bisphosphonates (unpublished original data).

Overview image of the bone surface showing numerous dead osteocytes (A). Magnification of the overview image shows that in the case of non-living osteocytes, the lacuno–canalicular network (B) and the integrity of the osteocyte body (C) are disrupted (cell wall disruption emphasized in inset of C).

# References

Arafat, M.T., Lam, C.X.F., Ekaputra, A.K., Wong, S.Y., Li, X., and Gibson, I. (2011). Biomimetic composite coating on rapid prototyped scaffolds for bone tissue engineering. *Acta Biomaterialia* 7(2)**,** 809-820. DOI: <https://doi.org/10.1016/j.actbio.2010.09.010>.

Huang, B., Caetano, G., Vyas, C., Blaker, J.J., Diver, C., and Bártolo, P. 2018. Polymer-Ceramic Composite Scaffolds: The Effect of Hydroxyapatite and β-tri-Calcium Phosphate. *Materials* [Online], 11(1).

Lam, C.X., Hutmacher, D.W., Schantz, J.T., Woodruff, M.A., and Teoh, S.H. (2009). Evaluation of polycaprolactone scaffold degradation for 6 months in vitro and in vivo. *J Biomed Mater Res A* 90(3)**,** 906-919. DOI: 10.1002/jbm.a.32052.

Laubach, M., Bessot, A., McGovern, J., Saifzadeh, S., Gospos, J., Segina, D.N., et al. (2023). An in vivo study to investigate an original intramedullary bone graft harvesting technology. *European Journal of Medical Research* 28(1)**,** 349. DOI: 10.1186/s40001-023-01328-8.
